# Supplementary material for: Transitional evolutionary forms in chasmosaurine ceratopsid dinosaurs: evidence from the Campanian of New Mexico
Source: PeerJ. 2020 Jun 5;8:e9251. doi: 10.7717/peerj.9251 (PMC7278894; doi:10.7717/peerj.9251)
Supplement: Supplemental Information 1 — Additional materials and methods including geological setting, and review of fossil materials, including discussion of specimen morphology. [file peerj-08-9251-s001.doc]

**SUPPORTING INFORMATION 1**

Additional materials and methods including geological setting, and review of fossil materials.

# Supplemental geology and methods

## Geological Setting

Campanian sediments of the San Juan Basin record transition from the marine Lewis Shale through the shallow marine Pictured Cliffs Sandstone into the nonmarine Fruitland and Kirtland formations (Bauer, 1916; Hunt et al., 1992; Lucas et al., 2006). The Fruitland Formation rests conformably upon the Pictured Cliffs Sandstone, and comprises 40–90 m of sandstones, occasional mudstones, and extensive thick coals deposited in a coastal plain setting. The overlying Kirtland Formation is ~260–360 m thick, and is less sandy, with only occasional thin coals, being similarly deposited on a coastal plain.

The stated variation in thickness of the Fruitland and Kirtland is in part due to the general thinning of San Juan Basin sediment fill towards the southeast, but also because many different definitions of the Fruitland and Kirtland Formations have been offered since their original description by Bauer (1916; S3). Mainly this concerns the definition of the lower formational contact between the Fruitland and Kirtland Formations, and the upper contact between the Kirtland and Ojo Alamo Formations. The result is a complex and confusing stratigraphic nomenclature, which is further complicated by the fact that geological maps of the San Juan Basin do not all use the same formational definitions, and commonly vary depending on the scale of the maps used, and when they were produced. However, historical stratigraphic usage must be understood if the stratigraphic positions of historical specimens of cf. *Pentaceratops* are to be determined, as most were collected from a stratigraphic zone considered as either the upper Fruitland Formation, or lowermost Kirtland Formation.

### Fruitland - Kirtland formational boundary

Three definitions of the boundary between the Fruitland and Kirtland formations are commonly encountered in the literature (Fig. S3), concerning either the highest economic coal, or a horizon known as the Bisti Bed sandstone. In the original definition, Bauer (1916) described the boundary between the Fruitland and Kirtland formations as transitional, and chose to place the formational boundary at the top of a consistent sandy interval (later named the Bisti Bed sandstone; Hunt and Lucas, 1992; Lucas et al., 2006). This definition was used in Bauer's 1916 geological map of the San Juan Basin, and in the current 250k map of the Shiprock quadrangle that covers most of the San Juan Basin (USGS I-345; O'Sullivan and Beikman, 1963).

Fassett and Hinds (1971) redefined the Fruitland - Kirtland formational boundary as occurring at the top of the uppermost "economic" coal bed, which is ~20–30 m stratigraphically lower than the definition of Bauer (1916). This also effectively restricted the Fruitland Formation to the unfossiliferous coal-zone, which is potentially confusing since most of the historical *Pentaceratops* specimens were cited as having been collected in the Fruitland Formation (see Supporting Information). Fassett and Hinds' (1971) definition was used to produce the currently available 24k USGS geological maps of the area (e.g. O'Sullivan et al., 1979; Weide et al., 1979) and most later maps (100k and 250k) are dependent on these for data.

The coal-based definition of Fassett and Hinds (1971) was criticized by Hunt and Lucas (1992) because of lateral inconsistency of the coals, and because thin coal horizons in the Kirtland Formation might be misinterpreted to be part of the Fruitland Formation, leading to confusion. Indeed, this error occurred during stratigraphic placement of the radiometric dates recovered by Fassett and Steiner (1997; see later), and has led to major inconsistencies in the mapping of the Fruitland - Kirtland boundary in at least some of the 24k USGS maps (for example, see Burnham Trading Post quadrangle; Scott et al. 1979). Hunt and Lucas (1992) therefore proposed using the base of the Bisti Bed sandstone as the Fruitland - Kirtland formational boundary, which is closer to Bauer's original (1916) meaning. Finally, Hunt and Lucas (2003) subdivided the Fruitland Formation into two members: the Neh-nah-ne-zad Member (13–30 m thick), comprising the almost completely unfossiliferous lower portion with economic coals (effectively equivalent to the less inclusive "Fruitland Formation" of Fassett and Hinds, 1971); and the Fossil Forest Member (12–25 m thick), comprising the fossiliferous mudstones and sandstones immediately underlying the Kirtland Formation.

### Kirtland Formation, internal stratigraphy

After redefining the Fruitland - Kirtland Formation contact as occurring at the base of the Bisti Bed, Hunt and Lucas (1992) formalized the members of the Kirtland Formation, renaming the lower shale member, Farmington Sandstone Member, and upper shale member (Bauer, 1916), as the Hunter Wash Member, Farmington Member, and De-na-zin Member, respectively. It is worth noting that, although all members thin to the southeast, the Farmington Member is especially variable in thickness, being up to ~140 m thick in the northern part of the basin, but pinching out entirely to the southeast at Ahshislepah Wash (Bauer, 1916). This accounts for differences that are sometimes encountered between published measured sections. It is important to note that Hunt and Lucas (1992) also chose to follow Baltz et al. (1966; Fig. S3) in including the dinosaur-bearing part of the overlying Ojo Alamo Formation (the Maastrichtian Naashoibito Member) as the uppermost member of the Kirtland Formation. This was later rescinded (Sullivan et al., 2005; Lucas et al., 2006; Fig. S3).

### Age

A series of five Ar / Ar radiometric dates for the Fruitland and Kirtland formations were published by Fassett and Steiner (1997). The lithostratigraphic positions of these dated horizons were later revised (Sullivan et al., 2005; Sullivan and Lucas, 2006) such that ashes 2 and 4, previously considered to occur in the Fruitland Formation, were shown to actually occur in the Kirtland Formation (stratigraphic positions of the other ashes were not affected). The radiometric dates of Fassett and Steiner (1997) were recalibrated (Fowler, 2017; Ar / Ar standard and decay constant pairing of Kuiper et al., 2008; and Min et al., 2000; respectively) to show the Fruitland through Kirtland formations were deposited between 76.0 and 73.4 Ma (see Figs. 1, 2). Note that previous recalibrations of these dates (Roberts et al., 2013) used incorrect Ar / Ar standards and decay constants, which consequently yielded ages ~1 m.y. too young (see Fowler, 2017, for details).

Magnetostratigraphy of the Fruitland and Kirtland formations has been controversial, although there is now a general consensus. The Fruitland and Kirtland formations are mostly of normal polarity, correlated to the upper part of C33n (79.900–74.309 Ma; Ogg, 2012). Stratigraphic placement of the C33n - C32r boundary within the San Juan Basin is slightly variable between workers. Lucas et al. (2009) place the C33n - C32r boundary in the lowermost De-na-zin Member, whereas Fassett (2009) places it in the upper part of the Farmington Member. Regarding the current study, there is little meaningful difference between these interpretations.

### Maps and stratigraphic positioning of San Juan Basin fossils

To assess stratigraphic position of San Juan Basin specimens (Fig. 2), locality data was plotted in Google Earth with geologic and topographic maps superimposed on the aerial photographs (Fig. 1). The locality coordinates of recalibrated radiometric dates were also plotted (Fassett and Steiner, 1997; Sullivan et al., 2005; Sullivan and Lucas, 2006; Fowler, 2017). No geological maps are available that use the current stratigraphic definitions of the Fruitland and Kirtland Formations (Sullivan et al., 2005; Lucas et al., 2006), so the main area of the geologic map (Fig. 1) is traced from the current 250k map of the Shiprock quadrangle (USGS I-345; O'Sullivan and Beikman, 1963) which uses the definitions of Bauer (1916) and covers most of the Fruitland and Kirtland Formation outcrop in the San Juan Basin. Unfortunately, the southeastern portion of the basin (Ahshislepah, Kimbeto, and Tsosie washes) is not covered by a geological map that uses the Bauer (1916) definitions. This issue concerns only the Fruitland-Kirtland boundary in Ahshislepah Wash, which contains the type locality (SMP Loc. 281; "Denver's blowout") of *Navajoceratops sullivani* (Fig. 1A), and some specimens collected by Charles H. Sternberg in the 1920s. To reconcile this, a tentative Fruitland-Kirtland boundary (sensu Bauer, 1916) was plotted based on aerial photography and the position of a prominent sandstone bluff (SMP Loc. 396; "Bob's bloody bluff") present ~0.7 km west-southwest of SMP Loc. 281. This was previously considered to represent the Bisti Bed sandstone (Sullivan, 2006), but is now thought to be slightly above the Bisti Bed (Sullivan pers. comm. 2019), based on discovery ~1km SE (and slightly lower in section) of a thick brown resilient sandstone exposed by channel weathering, which is much more like the type Bisti Bed.

Specimen locality data was retrieved either from the published literature or from institutional specimen databases. Details specific to the placement of individual specimens can be found in the "Fossil Materials" (below), and in Supporting Information 1. Some localities were historically only recorded as low-resolution Township / Range data, and these are therefore plotted as squares depicting the area of collection.

Relative stratigraphic position of specimens was inferred from the map by position relative to geological unit boundaries, topography, and trigonometric calculations based on a dip of 1–3° basinwards (northeast; Bauer, 1916). Stratigraphic height of the *Navajoceratops* holotype (SMP VP-1500) locality (SMP loc. 281) was calculated by comparison between the locality and two marker horizons. The uppermost prominent coal of the Fruitland Formation occurs ~1800 m to the southwest, 12.1 m topographically lower (Weide et al., 1979). The Bisti Bed sandstone occurs slightly below the base of "Bob's Bloody Bluff" (SMP loc. 396; Sullivan, 2006) ~700 m west-southwest (equivalent to ~500m parallel to dip). Stratigraphic height of the *Terminocavus* holotype (NMMNH P-27468) locality (NMMNH L-3503) was calculated from difference in elevation between the locality and two radiometrically dated horizons. Ash 2 (75.02 ± 0.13 Ma) occurs 3000 m southwest, and 30 m lower topographically. Ash 4 (74.57 ± 0.62) occurs 2200 m northeast, and 9 m higher topographically (ash dates recalibrated from Fassett and Steiner, 1997, by Fowler, 2017)

Bauer (1916) shows the Kirtland Formation as only 830 ft (253 m) thick in the Ahshislepah section (Meyers Creek of Bauer's usage), compared to a thickness of 1031 ft (314 m) at Hunter Wash (see map, Fig. 1). This does not affect interpretation of stratigraphic relationships in this current work, but should be noted by future researchers who might be working elsewhere in the San Juan Basin, in case specimen stratigraphic positions are cited as the number of metres above or below Kirtland formational contacts.

### Stratigraphic terminology

Through necessity, colored geological units on the map (Fig. 1) use formational boundaries defined by Bauer et al. (1916), but otherwise the map and manuscript follow the most recent terminology of Sullivan et al. (2005), and Lucas et al. (2006; see Fig. S3). Other than the names of the individual members, the only difference between Bauer (1916) and the most recent definitions are that Bauer places the Fruitland - Kirtland boundary at the top of the Bisti Bed sandstone, whereas the current definition places it at the base.

### Interbasinal correlation

Interbasinal correlation is necessary to ascertain the relative stratigraphic positions of chasmosaurine taxa that occur in different sedimentary basins across the Western Interior (e.g. *Utahceratops gettyi*, Kaiparowits Formation, Utah; Sampson et al., 2010). Correlating between sedimentary basins is fraught with problems, and depends upon accurate chronostratigraphic and biostratigraphic data being available. In this work the chronostratigraphic framework laid out in Fowler (2017) is used, which integrates recalibrated radiometric dates with other chronostratigraphic data (e.g. biostratigraphy, magnetostratigraphy) derived from The Geologic Time Scale 2012 (Gradstein et al., 2012). All radiometric dates are calibrated to the standard - decay constant pairing of Kuiper et al. (2008) and Min et al. (2000).

## Angle measurements

The angle formed at the midline of the lateral rami of the posterior bar was reconstructed by drawing lines along the midpoint of the rami on digital photographs in Adobe Photoshop CS2. Midpoint lines were originally plotted by drawing a line perpendicular to width midpoints on each ramus, but the results were inaccurate (due to distortion, asymmetry, and missing parts of the fossils), so lines were plotted by eye instead. The angle was measured using ImageJ 1.46r (Rasband, 1997–2014). See Figures S10 and S11.

## Measurements

Select measurements of the holotype materials of *Navajoceratops sullivani* and *Terminocavus sealeyi* can be found in Supplementary Figures S12, S13 and S14.

## Justification for placing new taxa into unique genera

For taxonomic stability and ease of reference, it was decided to place the new specimens into their own genera as otherwise they would have to be placed within either *Pentaceratops* or *Anchiceratops* (as either new species, or individuals of existing species), either of which would create problems.

An argument could be made that morphologic differences among the new specimens and those previously referred to *Pentaceratops sternbergii* are within that which might be expected of a single species, i.e. that they represent biological variation. However, each morphology is stratigraphically separated over a range of ~1–2 m.y. (with the possible exception of cf. *P. sternbergii*, and aff. *Pentaceratops* n. sp.), demonstrating that they should not be considered as belonging to the same population. Gradual enclosure of the parietal embayment between stratigraphically successive populations further supports their naming as separate taxa. The question is therefore whether the new taxa should be new species within *Pentaceratops*, or whether to erect new genera and species.

Placement of the new taxa as new species within *Pentaceratops* would be similar to the current treatment of stratigraphically separated *Triceratops* species (Scannella et al., 2014). This taxonomic approach would thus illustrate the hypothesis that we are observing evolution within an unbranching lineage, effectively chronospecies within an anagenetic lineage (as similarly suggested for *Triceratops*). However, this is problematic from a practical standpoint. *Triceratops* is a terminal taxon, and as such does not suffer from possible paraphyly emerging as a result of phylogenetic analysis; multiple species of *Triceratops* all exist within a single clade comprised only of the genus *Triceratops*, and not as stem taxa between separate genera (as is the case with the new taxa). To avoid paraphyly issues, it is current practice among dinosaur taxonomists to maintain monospecific genera. Thus, in order to maintain taxonomic stability, it is therefore preferable to place each morphology into its own genus. This is satisfactory so long as it is remembered that this choice of taxonomy does not necessarily imply vicariance events, and that an anagenetic lineage comprising *Utahceratops*, *Pentaceratops*, *Navajoceratops*, *Terminocavus*, "Taxon C", and *Anchiceratops* is an unfalsified hypothesis.

## Spelling of P. sternbergii

Lehman (1998) makes the technical suggestion that *Pentaceratops sternbergii* should be spelled as *Pentaceratops sternbergi*. This has not been followed by most subsequent workers (e.g. Dodson et al., 2004), thus here the original spelling of Osborn (1923) is used.

# Review of Fossil Materials

Here we review fossil materials of chasmosaurines relevant to the current study, including observations not previously published. Detailed descriptions are given for Campanian chasmosaurine specimens from the San Juan Basin, New Mexico, which are most comparable to the new specimens. Attention is also given to chasmosaurines from elsewhere in North America that preserve the parietal posterior bar, being therefore comparable to the newly described specimens, and can be included in the morphometric analysis. We also review problematic or fragmentary taxa (including many recently described taxa) that require reassessment or comment.

The following morphological features of the parietal are given special attention as they are most important when considering differences among chasmosaurines:

1. Presence and shape of an embayment on the posterior bar, including whether the embayment extends anterior to the posteriormost border of the parietal fenestrae.
2. Angle at which the lateral rami of the posterior bar meet medially.
3. Relative and absolute anteroposterior thickness of the posterior bar lateral rami from medial to lateral.
4. Number, shape, size, and orientation of epiparietals (typically 3), especially ep1, which is the most diagnostic.
5. Anteroposterior position of the maximum constriction of the median bar.
6. Development of lateral flanges of the median bar that invade the parietal fenestrae.
7. Development of the lateral bars to completely enclose the parietal fenestrae.

The precise stratigraphic position of specimens is also given special attention as this information is critical when assessing morphological change through time and its implications for the mode of evolution.

Ontogenetic development is assessed where relevant or possible, although this is largely limited to size, parietosquamosal frill texture, and shape and fusion of frill epiossifications.

### Institutional abbreviations

AMNH, American Museum of Natural History, New York; CMN, Canadian Museum of Nature, Ottawa (was NMC, National Museum of Canada); MNA, Museum of Northern Arizona, Flagstaff; NHMUK, Natural History Museum, London (was BMNH, British Museum Natural History); NMMNH, New Mexico Museum of Natural History and Science, Albuquerque; OMNH, Oklahoma Museum of Natural History, Norman; PMU, Paleontologiska Museet, Uppsala University, Sweden; ROM, Royal Ontario Museum, Toronto; SDNHM, San Diego Natural History Museum, California; SMP, State Museum of Pennsylvania, Harrisburg; TMP, Tyrrell Museum of Paleontology, Drumheller, Alberta; UALP, University of Arizona Laboratory of Paleontology; KUVP, University of Kansas, Lawrence; UMNH, Utah Museum of Natural History, Salt Lake City; UNM, University of New Mexico, Albuquerque; USNM, United States National Museum, Smithsonian Institution, Washington D.C.; UTEP, University of Texas at El Paso.

## Campanian San Juan Basin chasmosaurine specimens

### *Pentaceratops sternbergii* holotype AMNH 6325, and cf. *P. sternbergii* referred specimens AMNH 1624, 1625

The holotype of *Pentaceratops sternbergii* (AMNH 6325; Osborn, October 1923) was collected in the San Juan Basin by Charles H. Sternberg in 1922, and comprises a mostly complete skull that unfortunately lacks the diagnostic posterior bar of the parietal. A second near-complete skull (AMNH 1624, currently undescribed but figured as a line drawing by Lehman, 1993; 1998; and numerous photographs in Sullivan and Lucas, 2011) was collected by C.H. Sternberg in the San Juan Basin in June 1923 (*contra* Lull, 1933; see C.H. Sternberg's letters in Sullivan and Lucas, 2011; p. 455; see below), but similarly lacks the distal part of the parietal. Both of these specimens were reconstructed for display based on a third specimen (AMNH 1625, also undescribed but figured by Longrich, 2014) collected by C.H. Sternberg in 1923 (*contra* Lull, 1933; see below), which is a near-complete frill missing only the anterior ends of the parietal and squamosal; as such it can be directly compared with the new specimens described here. Osborn (1923) does not mention the existence of AMNH 1624 or AMNH 1625 (nor AMNH 1622, a set of brow horns), as they were not acquired by the AMNH until 1924 (see letters in Sullivan and Lucas, 2011). After the specimens were acquired by the AMNH, it appears likely that they were labeled as *P. sternbergii* in the AMNH collections, as Lull (1933) includes them in a list of *Pentaceratops sternbergii* specimens without any specific comment on them not being previously described or figured, nor listing characters that would permit referral.

**Stratigraphy**

As AMNH 6325 is the type specimen of *P. sternbergii*, it is clearly important to establish its provenance. All available data suggests that the holotype of *Pentaceratops sternbergii* (AMNH 6325), and probably the referred specimens AMNH 1624 and 1625 were collected from the Fossil Forest Member of the Fruitland Formation, probably from a sandstone in the middle of the Fossil Forest Member. In the original description (Osborn, 1923), the holotype (AMNH 6325) was described as having been collected from the Fruitland Formation, but no further details are given. This was later expanded upon by Lull (1933) who stated that AMNH 6325 was collected from 9 miles east of Tsaya (a trading post). Using this description, Rowe et al. (1980) suggest that the collection locality is "probably NW 1/4 T22N R11W", however, this defined area (Fig. 1H) has very little to no outcrop visible on Google Earth satellite images, consisting instead of flat scrubland. It is unlikely that vegetation cover and topography have changed significantly since the specimen was collected in 1922, so the locality suggested by Rowe et al. (1980) is probably incorrect. Hunt and Lucas (2003) suggest that the holotype might have been collected from one of the many old quarries (up to 12; Wolberg et al., 1988; only 5 described by Hunt and Lucas, 2003) located in the Fossil Forest study area (Fig. 1), which they point out is 8–9 miles northeast of Tsaya (i.e. mostly matching the descriptions of Lull, 1933; and Rowe et al., 1980), and from which it is generally accepted that C.H. Sternberg collected fossil material. All of the historical quarries described by Hunt and Lucas (2003) were excavated into a sandstone horizon approximately in the middle of the Fossil Forest Member of the Fruitland Formation. Furthermore, the Fossil Forest study area has little to no outcrop that occurs above the Bisti Bed (the lowermost member of the Kirtland Formation), such that specimens collected from this locality are all likely to have originated from the Fruitland Formation. The referred partial skull AMNH 1624 is described as having been "collected by CH Sternberg in 1923 from the Fruitland Formation, near Tsaya" (Lull, 1933). The diagnostic nearly complete frill (AMNH 1625) was "collected by CH Sternberg from the Fruitland Formation in 1924" (Lull, 1933). Rowe et al. (1980) acknowledge the existence of AMNH 1625 but state that they had not seen the specimen. Further information is provided in Sternberg's letters to the AMNH documented by Sullivan and Lucas (2011); in their appendices (p. 447), Sullivan and Lucas (2011) suggest that a ceratopsid frill found by C.H. Sternberg in the Fruitland Formation (mentioned by Sternberg in a letter to Prof. Carl Wiman of the Paleontologiska Museet at Uppsala University; dated 4th Sept 1921) is AMNH 1625. However, this is probably in error as all material collected during 1921 should have been shipped to Uppsala (the 1921 specimen is probably therefore one of the other pieces of ceratopsian frill that Sternberg sent to Uppsala, likely incomplete left squamosal PMU 24944 (was PMU R.220), as this is the only frill piece from the Fruitland Formation in the Uppsala University collection). In a later letter from Sternberg to W.D. Matthew of the AMNH (June 27th, 1923; in Sullivan and Lucas, 2011, p. 456), Sternberg refers to a newly discovered frill as "the entire distal margin of the crest and the central bar are entire, also part of both squamosals and margin of crest." Unfortunately, no locality details are given, but this accurately describes the preserved material of AMNH 1625. Indeed, Sternberg had found AMNH 1624 a few weeks previously (documented in his letter dated June 17th 1923; Sullivan and Lucas, 2011) and offered both specimens to the AMNH as a set. Based on this information, AMNH 1625 was presumably found and collected in 1923, not 1924 (as stated by Lull, 1933). Sternberg's 1923 letters were sent from Tsaya, but other than that no locality information is available for AMNH 1625, however it seems likely that he was working in the same area in which he discovered AMNH 1624 only weeks previously. Regardless, AMNH 6325, 1624, and 1625 are all described as having been collected in the Fruitland Formation.

**Morphology**

As AMNH 6325 (holotype, *Pentaceratops sternbergii*) lacks the posterior end of the frill, it cannot be compared with the new specimens described here. Some comparisons can be made between the new specimens and AMNH 1624 as it preserves the right posterolateral corner of the parietal (at its contact with the squamosal). AMNH 1625 is a nearly complete frill so can be fully compared with the new specimens. Morphological assessment of AMNH 1624 and 1625 is based on images and description provided in Lehman (1998), Sullivan and Lucas (2011), and Longrich (2014).

Specimen AMNH 1625 is as large or larger than other specimens previously referred to *Pentaceratops* (Fig. 7). The lateral rami of the posterior bar meet at an angle of 83° (Fig S10). The anteroposterior thickness of the posterior bar of AMNH 1625 is either relatively consistent mediolaterally, making it appear strap-like, or is actually thicker medially than laterally (also seen in *Utahceratops* referred specimen UMNH VP-16671 and coded as a new character by Longrich, 2014). This is unlike specimens of aff. *Pentaceratops* n. sp. (KUVP 16100, MNA Pl.1747, and NMMNH P-37880), *Navajoceratops*, and *Terminocavus*, where the lateral rami of the posterior bar are anteroposteriorly narrower medially than laterally. The lateral rami of AMNH 1624 are also unexpanded anteroposteriorly at the contact with the squamosal. Each lateral ramus of the posterior bar is bent at an obtuse angle at the ep2 locus. This is not observed in specimens referred to aff. *Pentaceratops* n. sp. (KUVP 16100; MNA Pl.1747; NMMNH P-37880), however, an obtuse angle at the ep2 locus is present in *Utahceratops gettyi* (referred specimen UMNH VP-16671, and probably the holotype UMNH VP-16784; Sampson et al., 2010), and also possibly *C. russelli* referred specimen TMP 1999.55.292 (Longrich, 2010). A similarly angular posterior bar is also seen in *C. russelli* referred specimen CMN 8803 (Sternberg, 1940; Longrich, 2010), and *Agujaceratops* specimen UTEP P.37.7.065 (Lehman, 1989), although in these specimens the angular deflection occurs at the locus labeled as ep1. It is unclear if this angular shape for the posterior bar has taxonomic significance, or might simply represent individual variation.

The median bar of AMNH 1625 is narrow and strap-like with the point of maximum constriction occurring approximately half way along its length. The ventral side of AMNH 1625 was not figured by Longrich (2014) so it is unknown whether or not there are any lateral flanges that extend into the fenestrae; however, the narrowness of the bar suggests that they are not present. The median bar of AMNH 6325 is similarly narrow and strap-like, but the specimen is encased in a wall mount and so further comparisons are not possible.

The parietal lateral bars of AMNH 1625 are either broken, imperceptibly fused to the squamosal bar, or are simply less encompassing of the parietal fenestrae than in specimens of aff. *Pentaceratops* n. sp. (and later chasmosaurines). The degree to which the parietal fenestrae are entirely enclosed within the parietal is noted as variable even between left and right sides of the same individual in *Chasmosaurus belli* (Godfrey and Holmes, 1995). However, given that in more derived chasmosaurines the parietal fenestrae are completely enclosed by a thick parietal lateral bar (e.g. *Anchiceratops*, “*Torosaurus*”), then variability in this character is probably phylogenetically important (see main text).

AMNH 1625 is one of the few frills to exhibit all epiparietals fused to the posterior bar. Although the typical six epiparietals are present, the lateralmost epiparietals (ep3) on both sides straddle the parietal-squamosal contact such that under some classification systems (e.g. Sampson et al., 2010) they might be considered as "eps" rather than ep3. Most other specimens of cf. *P. sternbergii* do not preserve an epiossification fused to locus ep3 (although one presumably existed there in life). The exception is AMNH 1624, which has the lateralmost part of the parietal preserved and an ep3 that abuts but does not appear to straddle the parietal-squamosal contact. *Terminocavus* preserves a fused ep3 on the left side which does not straddle the squamosal-parietal contact. This suggests that, as in *Triceratops*, the “eps” is an individually variable feature (perhaps strongly influenced by ontogeny), and might not be a reliable character for phylogenetic analysis. However, it remains an interesting source of variation within a population.

Ep1 is interesting in AMNH 1625 as on the left side it projects anterodorsally (as seen in c.f. *Pentaceratops* sp. KUVP 16100 and MNA Pl.1747) but on the right side only dorsally. Longrich (2014) suggests that this is a preservational artifact, but it is hard to imagine a burial process where the left ep1 remains as normal, whereas the right side is distorted to deflect dorsally, especially in this case where there is otherwise no unusual distortion elsewhere on the frill. We therefore interpret the difference in orientation of left and right ep1 in AMNH 1625 as genuine, which has repercussions for the validity of *P. aquilonius* (Longrich, 2014; see later).

In AMNH 1625 ep2 projects posteriorly, as in *Utahceratops* referred specimen UMNH VP-16671, but unlike c.f. *Pentaceratops* sp. MNA Pl.1747, *Navajoceratops,* and *Terminocavus*, in which ep2 projects medioposteriorly. Ep2 is damaged in KUVP 16100, but would appear to be intermediate in orientation between that of AMNH 1625 and MNA Pl.1747, being mostly posteriorly oriented, but slightly medially deflected (Rowe et al., 1980; Lehman, 1998). In the new specimen c.f. *P. sternbergii* NMMNH P-37880, ep2 is similarly unfused, but the ep2 locus is oriented either posteriorly or slightly medioposteriorly (see later).

In AMNH 1625 (and almost certainly AMNH 1624), the angular shape of the posterior bar means that the posteriormost epiparietal is ep2, with ep3 either subequal or anterior to ep2. This condition is also seen in *Utahceratops* specimens UMNH VP 16784 (holotype) and UMNH VP 16671. However, in aff. *Pentaceratops* n. sp. specimens MNA Pl.1747 and KUVP 16100, *Navajoceratops* holotype SMP VP-1500, and *Terminocavus* holotype NMMNH 27468, ep3 is the posteriormost locus.

In summary, cf. *P. sternbergii* referred specimen AMNH 1625 exhibits morphology intermediate between *Utahceratops* referred specimen UMNH VP-16671 and specimens referred to aff. *Pentaceratops* n. sp. (KUVP 16100 and MNA Pl.1747).

### aff. *Pentaceratops* n. sp.; KUVP 16100

KUVP 16100 was collected in 1966 by the University of Kansas Museum of Natural History and comprises a skull with frill. Most of the skull has not been described, but the critical posteromedial area of the parietal was briefly described and figured by Rowe et al. (1980), and Lehman (1998), who both referred it to *Pentaceratops sternbergii*. The specimen has recently been completely reprepared and is currently being described (J. Fry, pers. comm.).

**Stratigraphy**

Rowe et al. (1980) report that KUVP 16100 was collected from the upper part of the Fruitland Formation in the SW 1/4, Sec. 34, T24N R13W, Hunter Wash, New Mexico. This is refined to the SE 1/4 of the SW 1/4 of the SW 1/4, Sec. 34, T24N R13W (Fig. 1E; RM Sullivan, pers. comm. 2015). This quarter-mile square plots entirely within the uppermost part of the Fruitland Formation in the USGS 100k bedrock geology map (O'Sullivan and Beikman, 1963), or alternatively in the lowermost part of the Kirtland lower shale (Hunter Wash Member) in the USGS 24k surficial geology map (O'Sullivan et al., 1979). From this, it can be inferred that KUVP 16100 occurs stratigraphically between the uppermost coal of the Fruitland Formation and the top of the Bisti Bed sandstone (sensu Lucas et al., 2006). This is supported by reference to aerial photography of the area in Google Earth, which reveals extensive dark red-brown sandstones matching the description of the Bisti Bed sandstone (Hunt and Lucas, 1992; Lucas et al., 2006). Finally, this specific area was illustrated in an aerial photograph by Hunt and Lucas (1992) and identified as outcrop of the Fossil Forest Member of the Fruitland Formation and the Bisti Bed sandstone (indeed, this is the type area for the Bisti Bed; Hunt and Lucas, 1992). From this information it is considered that KUVP 16100 was collected from the Fossil Forest Member of the Fruitland Formation.

**Morphology**

KUVP 16100 is currently under study (J. Fry, pers. comm.) so discussion of its morphology is brief and limited to the parietal, that being the element relevant to description of the new specimens, and the only element previously figured or described (in ventral view by Rowe et al., 1980; in dorsal view by Lehman, 1998). The figured portion of the parietal comprises a complete posterior bar, and the posterior ends of the lateral bars. A median bar is figured by Rowe et al. (1980), but may not be correctly identified, and so it will not be further discussed here.

The lateral rami of the posterior bar meet at an angle of 87° forming a deep embayment that extends anteriorly slightly further than the posteriormost edge of the fenestrae (Fig. S10). The posterior bar is of similar anteroposterior thickness from the midpoint to the lateral edges, although the lateral edges are slightly anteroposteriorly thickened at the apex. With regards to anteroposterior thickness, KUVP 16100 is intermediate between AMNH 1625 (where the posterior bar is of uniform thickness) and MNA PL.1747 (which is significantly thicker at the lateral margins). This represents an interesting trend in San Juan Basin chasmosaurines where the anteroposterior thickness of the posterior bar changes stratigraphically, culminating in the condition seen in *Navajoceratops* and *Terminocavus* (see main text).

Only the posteriormost ends of the parietal lateral bars are figured by Rowe et al. (1980), but this at least demonstrates that the lateral bars are more substantial than in *P. sternbergii* specimen AMNH 1625. However, the extent to which they might enclose the parietal fenestrae must await full description of the specimen.

KUVP 16100 preserves loci for three epiparietals on each side, with some epiparietals fused in place (Rowe et al., 1980; Lehman, 1998; J. Fry, pers. comm.). Both ep1 are recurved to point anterolaterally (Lehman, 1998) as seen in other specimens of *Pentaceratops*. Lehman (1998) illustrates fused epiparietals at locus ep2, but it would appear that this is only visible on the right side (J. Fry, pers. comm.). Ep3 loci do not bear fused epiparietals and are positioned immediately lateral to ep2.

The parietal of KUVP 16100 exhibits characters consistent with a subadult status in that only ep1 has fused epiossifications. Unfused epiossifications at loci ep2 and ep3 are supportive of a subadult status (based on ontogenetic analysis of *Triceratops*; Horner and Goodwin, 2006). Ontogenetic assessment of elements other than the parietal await full description of the specimen by J. Fry.

### aff. *Pentaceratops* n. sp.; MNA Pl.1747

MNA Pl.1747 was collected in 1975 by the Museum of Northern Arizona. It comprises a mostly complete skull, including the diagnostic posterior bar of the parietal. The specimen has never been completely described, although partial descriptions have been given by Rowe et al. (1980), and Lehman (1993), and a full description is in preparation (J. Fry, pers. comm.).

**Stratigraphy**

The collection locality of MNA Pl.1747 has been previously misreported and is corrected here. Rowe et al. (1980) give the locality as the NW 1/4, SW 1/4, Sec. 28, T24N, R13W; ~5km east of the Burnham Trading Post (destroyed by fire in 1978). However, this is in error as the township/range coordinates are inconsistent with the locality description. The township range coordinates plot a one-sixteenth-mile square within the southwesternmost part of the Bisti-De-na-zin wilderness, along Hunter Wash, ~2.1 km ENE of the old Bisti trading post (Fig. 1). By comparison, the Burnham trading post was located ~25 km northwest of this position, in Brimhall Wash (Fig. 1). A locality map on file at the Museum of Northern Arizona resolves this problem, showing that MNA Pl.1947 was collected from section 4, T24N R15W, ~5 km southeast of Burnham trading post (Fig. 1F). The locality data of Rowe et al. (1980) should therefore be disregarded. Qualified researchers can request more precise locality data from the Museum of Northern Arizona.

The locality of MNA Pl.1747 plots within the upper part of the Fruitland Formation in the USGS 100k bedrock geology map (O'Sullivan and Beikman, 1963; Fig 1), and in the lowermost part of the Kirtland lower shale (Hunter Wash Member) in the USGS 24k surficial geology map (Scott et al., 1979). From this, it can be inferred that MNA Pl.1747 occurs stratigraphically between the uppermost coal of the Fruitland Formation and the top of the Bisti Bed sandstone (sensu Lucas et al., 2006).

**Morphology**

MNA Pl.1747 is currently under study (J. Fry, pers. comm.) so discussion of its morphology is brief and mostly limited to the parietal, that being the element relevant to description of the new specimens, and the only element previously well figured or described (Rowe et al., 1980; Lehman, 1993).

The parietal of MNA Pl.1747 is complete and well preserved. The posterior bar is anteroposteriorly narrow medially, but thickens laterally (although this may be partly an artifact of fusion of epiparietal 3; see later). The lateral rami of the posterior bar meet at an angle of 88° (Fig. S10) forming a deep embayment that extends slightly (4 cm) more anteriorly than the posteriormost edge of the fenestrae (Rowe et al., 1980).

The median bar is narrow and strap-like with the point of maximum constriction occurring approximately half way along its length. The ventral side was figured by Rowe et al. (1980) and does not exhibit the lateral flanges present in Taxon C (and to a lesser extent in *Navajoceratops* and *Terminocavus*; see main manuscript).

The parietal lateral bars are figured by Rowe et al. (1980), and completely enclose the fenestra on the left side. The right side is incompletely preserved.

MNA Pl.1747 preserves loci for three epiparietals on each side, with ep1 and ep2 fused on both sides (Rowe et al., 1980; Lehman, 1993). Both ep1 are recurved such that their tips point anterolaterally (Lehman, 1993). Both ep2 are preserved attached to the posterior bar (the suture is still visible), and are oriented dorsomedially, with their distal tips separated by only 12cm (Rowe et al., 1980). The medial edges of both ep2 are twisted slightly, being directed anterodorsally. Ep3 loci are described by Rowe et al. (1980) and Lehman (1993) as not bearing a fused epiparietal. This is how the specimen is reconstructed in our figures. However, J. Fry (pers. comm.) has suggested that ep3 is actually fully fused to the posterior bar on both sides, being distinguished only by a barely perceptible textural change (also implied in the reconstruction provided by Forster et al., 1993). If true, it would mean that the shape of the posterior bar would be very close to that of KUVP 16100 and NMMNH P-37880, but less similar to *Navajoceratops*. This issue will remain open until the complete redescription of MNA Pl.1747 is published by J. Fry.

MNA Pl.1747 exhibits characters consistent with an adult or subadult status (in comparison to ontogenetic indicators in *Triceratops*; Horner and Goodwin 2006; 2008). Epiparietals ep1, ep2, and possibly ep3 are fused to the parietal. In contrast only the anterior episquamosals are fused (see Rowe et al., 1981). Ontogenetic assessment of other elements awaits full description of the specimen by J. Fry.

### aff. *Pentaceratops* n. sp.; NMMNH P-37880

NMMNH P-37880 comprises ~50% of the right ramus of a parietal posterior bar (Fig. S15). Although incomplete, it preserves enough morphology to be compared to other chasmosaurine parietals, and so is figured and described here for the first time.

**Stratigraphy**

NMMNH P-37880 was collected in 1998 by Paul Sealey from NMMNH locality L-4010 and has been plotted from accurate UTM coordinates held on file at NMMNH (Fig. 1D). L-4010 occurs in the base of a white silty sand at the southwestern end of the south branch of Hunter Wash. The locality plots high in the Fruitland Formation, close to the boundary with the Kirtland Formation on the USGS 100k bedrock geology map (O'Sullivan and Beikman, 1963; Fig 1), and within the lowermost part of the Kirtland lower shale (Hunter Wash Member) on the USGS 24k surficial geology maps (O'Sullivan et al., 1979). It is listed as having been collected from the Fruitland Formation, and the map plot suggests that it occurs high in the Fossil Forest Member.

**Morphology**

NMMNH P-37880 comprises ~50% of the right ramus of the parietal posterior bar, and derives from an individual approximately the same size as KUVP 16100 and MNA Pl.1747 (Fig. 7). The posterior bar is embayed medially, but is broken and missing the midline. Similarly the lateralmost edge of the posterior bar is broken, such that the articulation surface with the squamosal is missing. No epiparietals are fused to the posterior bar, although two or three shallow bumps (loci) project from the posterior margin to which epiparietals are presumed to have attached. The medialmost locus (?ep1) is deflected slightly dorsally and divided into three smaller bumps, reminiscent of the irregular shape of ep1 in *Utahceratops* UMNH VP 16671. The next lateralmost locus (?ep2) is directed posteriorly, lying within the plane of the parietal itself. Further lateral to ?ep2, the posterior border is raised to a bump which would appear to be ep3, then is broken. In other specimens of *Pentaceratops*, only ep1 is typically dorsally deflected, supporting the identification that the medialmost locus of NMMNH P-37880 is locus ep1. However, it is possible that ep1 is not preserved on this portion of the parietal, and that instead the medialmost preserved locus is in fact ep2 (with the lateral most locus ep3). The posterior edge within the median embayment is slightly curved dorsally, perhaps indicative of attachment of ep1 more medially, but which is not preserved. The posterior bar is anteroposteriorly thicker laterally, and narrows medially (as seen in KUVP 16100, MNA Pl. 1747, *Navajoceratops*, and *Terminocavus*).

The large size of the specimen might be taken as an indication of maturity. However, as no epiparietals are fused to the posterior bar (which is an indication of subadult status in *Triceratops*; Horner and Goodwin, 2006; Scannella and Horner, 2010), it is likely that the specimen should be considered a subadult or younger. This is supported by the surface texture which exhibits only a few shallowly indented blood vessel grooves, and instead preserves vestiges of a long grain texture, which has been associated with immaturity in ontogenetic studies of centrosaurines and *Triceratops* (Brown et al., 2009; Scannella and Horner, 2010).

NMMNH P-37880 is here referred to aff. *Pentaceratops* n. sp., based on the presence of the following characters exhibited by MNA Pl.1747 and KUVP 16100 (see main text): Arches of the M-shaped parietal posterior bar rounded rather than angular. Lateral rami of the parietal posterior bar become more anteroposteriorly broad from medial to lateral, rather than being uniform thickness.

### aff. *Pentaceratops* sp. ("*Titanoceratops ouranos*") OMNH 10165

OMNH 10165 was collected in July 1941, probably from the Fruitland Formation, New Mexico, by Wann Langston, Jr., D.E. Savage, and J.W. Stovall for the Oklahoma Museum of Natural History (Lehman, 1998). The original description of OMNH 10165 (Lehman, 1998) referred it to *Pentaceratops sternbergii*. However, Longrich (2011) recently redescribed it as the holotype of the new taxon *Titanoceratops* *ouranos*. Longrich (2011) further suggested that *Titanoceratops* is much more derived than *Pentaceratops*, representing a very early-occurring representative of the *Triceratopsini*, which are otherwise only known from the Maastrichtian [*Eotriceratops* + "*Torosaurus*" + *Triceratops*]. However, many of the characters later used by Longrich (2011) to diagnose *Titanoceratops* were originally noted by Lehman (1998) as subject to a high level of intraspecific variability, and thus not useful in species-level discrimination among chasmosaurines. Furthermore, recent work on chasmosaurine ontogeny has highlighted that characters used by Longrich (2011) to distinguish "*Titanoceratops*" from *Pentaceratops* are known to change ontogenetically in other chasmosaurines, notably characters: 1, large size; 3, anterior squamosal epioccipitals low and with a dorsal ridge; 6, extensive cornual sinuses; 8, postorbital horns strongly curved anteriorly; 9, postorbital horns extremely elongate; 14 premaxillae extremely elongate (Lehman, 1998; Farke, 2006; Horner and Goodwin, 2006; Horner and Goodwin, 2008; Scannella and Horner, 2010; Scannella et al., 2014). It is therefore possible (if not probable) that differences between OMNH 10165 and other specimens referred to *Pentaceratops* might be due to OMNH 10165 being an especially large and old individual, as per its original description (Lehman, 1998). A reanalysis of this specimen is currently being undertaken by other researchers (H. N. Woodward, pers. comm. 2014), so its morphology will not be addressed in detail here.

**Stratigraphy**

The collection locality of OMNH 10165 is suggested to be 40 miles south of Aztec, New Mexico, ~5 miles south of former headquarters of Wood Ranch, in headwaters of Coal Creek (Longrich, 2011; citing Lehman, 1998, and Wann Langston's unpublished field notes). However, Hunt & Lucas (2003; not cited in Longrich, 2011) express some doubts, as Wolberg et al. (1988) relocated the Wood Ranch headquarters as being in the SE 1/4, NW 1/4, sec. 36, T24N R12W, therefore the location of the OMNH specimen quarry should be in sec. 25, T23N, R12W, which has no outcrop. Hunt & Lucas (2003) therefore suggest that the section immediately north of this (sec. 24, T23N, R12W) could be the true location of the quarry; it is this section that contains the famous "Fossil Forest" Study Area (see Fig. 1; Fossil Forest Member of the Fruitland Formation; Hunt and Lucas, 2003; Lucas et al., 2006) which has a number of large historical quarries from which the specimen might have been extracted (Hunt and Lucas, 2003).

Note that in the description of OMNH 10165 by Longrich (2011) the relative position of the radiometric date 74.11 Ma (unrecalibrated, Ash 4 of Fassett and Steiner, 1997) is misplotted as occurring at the top of the Fruitland Formation. This was indeed the original described stratigraphic position (Fassett and Steiner, 1997), however, the sample horizon was relocated by Sullivan and Lucas (2006) and found to occur relatively high in the Hunter Wash Member of the Kirtland Formation, rather than the uppermost Fruitland Formation (confirmed here by plotting the coordinates given by Fassett and Steiner, 1997; see Fig. 1). Thus the cited age range for OMNH 10165 (Longrich, 2011) is incorrect regardless of whether or not it is possible to assert a collection locality.

**Morphology**

The type specimen of *Titanoceratops* OMNH 10165 lacks the diagnostic posteromedial part of the parietal, therefore it is not possible to fully compare the specimen with other San Juan Basin chasmosaurines. However, it can be noted that the parietal median bar of OMNH 10165 is relatively narrow and lacks lateral flanges; i.e. it is unlike the condition seen in Taxon C (NMMNH P-33906), and all later chasmosaurines (*Anchiceratops*, *Arrhinoceratops* and all Triceratopsini, sensu Longrich, 2011). This is consistent with the suggestion that the specimen was collected from the Fruitland Formation (see above), and it is likely that it pertains to the same taxon as other specimens from the Fruitland, i.e. cf. *Pentaceratops sternbergii* (AMNH 1624, 1625, 6325) or cf. *P*. n. sp. (MNA Pl.1747; KUVP 16100; NMMNH P-37880).

It is further noted here that Longrich (2011) proposes that the scapula of OMNH 10165 is different from *Pentaceratops*, but this is based on the postcranium (PMU R.268; now recatalogued as PMU 24922; Kear pers. comm. 2015) described by Wiman (1930), and attributed to *P. fenestratus*. This postcranium (with an associated dentary) is not diagnostic to species, and has not been demonstrated to contain overlapping diagnostic material with the skull attributed to *P. fenestratus*, nor any other chasmosaurine specimen from the Fruitland or Kirtland Formations, with the exception of OMNH 10165. This postcranium cannot therefore be referred to *Pentaceratops* and should be considered as Ceratopsidae sp. Hence any differences between the postcranial skeletons cannot be used to infer that OMNH 10165 is different from *Pentaceratops*.

### *"Pentaceratops* *fenestratus*", holotype PMU 24923

The holotype of *Pentaceratops fenestratus* (Wiman, 1930) is a near-complete skull (PMU 24923; number reassigned from PMU R200; Kear pers. comm. 2015) collected by Charles H. Sternberg in 1921 from the Kirtland Formation, New Mexico (Sullivan and Lucas, 2011). The skull was designated a new species of *Pentaceratops* based on fenestration of the squamosal (Wiman, 1930). This was later deemed pathological by Lull (1933), as such extraneous squamosal fenestrae are relatively common in ceratopsids (e.g. see Tanke and Rothschild, 2002) and may be related to thinning of the squamosal through ontogeny rather than being strictly pathological. However, Lull (1933) never went so far as to sink *P. fenestratus* into *P. sternbergii*; indeed, Lull commented that *P. fenestratus* was probably a distinct form (based on it being recovered from younger sediments than *P. sternbergii),* just that he was not convinced by the diagnosis. More recently, Rowe et al. (1980) similarly discussed the diagnosis of Wiman (1930), but did not synonymize the species. However, more recently *P. fenestratus* has generally been treated as a junior synonym of *P. sternbergii* by most researchers (e.g. Dodson et al., 2004).

**Stratigraphy**

In his letters to Prof. Carl Wiman (Paleontologiska Museet at Uppsala University, which purchased the specimen; Sullivan and Lucas, 2011; p. 446), Sternberg specifically notes that PMU 24923 was collected from "what our geologists call the Kirtland Shales" and references Bauer (1916) and Gilmore (1919). If this is correct, then PMU 24923 was recovered from higher in section than other San Juan Basin specimens for which data is available (KUVP 16100; MNA Pl.1747; NMMNH P-37880; all confirmed as from the Fruitland Formation; AMNH 6325, 1624, 1625; reported as from the Fruitland Formation by Sternberg; see above). As such PMU 24923 may derive from a stratigraphic position in the Kirtland Formation equivalent to *Navajoceratops* or *Terminocavus*. Preservation (bone color and mineralization) of the PMU 24923 specimen is inconsistent with the specimen being derived from the stratigraphically higher De-na-zin Member (ie. the collection horizon of Taxon C).

**Morphology**

Morphological assessment is based on the description and figures of Wiman (1930) and photographs of specimens provided by R.M. Sullivan (SMP; NMMNH) and B. Kear (PMU). PMU 24923 is laterally compressed and slightly crushed such that the morphology of the posterior border of the parietal is difficult to ascertain. However, the diagnostic midline of the posterior bar is preserved and is more comparable to Kirtland Formation taxa *Navajoceratops* and *Terminocavus,* than to c.f. *Pentaceratops sternbergii* from the Fruitland Formation. The median embayment of the posterior bar is deep, notch-shaped and bordered by a raised rim, from which the paired epiparietals (locus ep1) project anterodorsally. Although the narrowness and depth of the notch may be affected by post-burial compression, it seems unlikely that this distortion is severe enough for the original morphology to have resembled *P. sternbergii*, and so it was probably more similar in life to *Navajoceratops* or *Terminocavus*. As preserved, the posterior bar is anteroposteriorly broad, unlike the narrow strap-like condition of Fruitland Formation c.f. *Pentaceratops* (AMNH 1625 and KUVP 16100) and more similar to *Terminocavus*. The morphology is otherwise mostly difficult to determine; although the parietal-squamosal contact is preserved, it is difficult to judge what the morphology of this area would be like as the bone is too distorted.

Similarity of PMU 24923 to *Navajoceratops* and *Terminocavus* is unsurprising since these specimens were all recovered from the Kirtland Formation. However, we concur with Lull (1933) in concluding that although *P. fenestratus* is probably distinct from *P. sternbergii*, distortion of the preserved material is too great to permit an accurate diagnosis. Either *Navajoceratops* or *Terminocavus* might be referable to the same taxon as PMU 24923, but it is impossible to demonstrate this based on current material.

### Other Uppsala specimens: PMU 24924

It is worth noting that during the 1920's, Sternberg collected a number of other chasmosaurine elements from the Fruitland and Kirtland Formations that were sent to Paleontologiska Museet at Uppsala University (Wiman, 1930; Sullivan and Lucas, 2011). Most of these specimens are probably not diagnostic to the same level as the posterior bar of the parietal. However, PMU 24924 (was PMU R.223; Kear, pers. comm. 2015) is an anterior part of the parietal preserving the median bar which is quite robust and bears small flanges directed laterally into the fenestrae, forming a broad T-shape in cross section (Fig. S16C). The flanges are not as well developed as in Taxon C from the De-na-zin Member, but are more developed than in *Pentaceratops sterbergii* and cf. *P.* n. sp., and similar to that observed in *Navajoceratops*. This is consistent with the collection locality which is recorded as being in the Kirtland Formation, rather than the Fruitland Formation (Wiman, 1930), and supports the hypothesis that the lateral flanges of the median bar become more prominent through time (see main text).

### Chasmosaurinae sp. NMMNH P-50000 (formerly UALP 13342)

NMMNH P-50000 (formerly UALP 13342) is a partial skull collected in 1975 by the University of Arizona, and is currently under study (J. Fry, S. G. Lucas, pers. comm.). The skull has a well preserved facial region, but lacks the frill and postorbital horns (although originally present, the right postorbital horn was apparently lost at some point prior to the specimen's transferal to NMMNH). Since it lacks a frill, it is not comparable to the new specimens described here, however, it is relevant because it was collected from high in section (see below) and because it was used to code character states for *Pentaceratops sternbergii* by Sampson et al. (2010).

**Stratigraphy**

Rowe et al. (1980) report that NMMNH P-50000 was collected from the SE 1/4 of the NE 1/4, Section 14, T24N R12W, Alamo Wash, New Mexico (see Fig. 1). This plots within the Farmington Sandstone Member of the Kirtland Formation using the USGS 100k bedrock geology map (O'Sullivan and Beikman, 1963; Fig. 1), or (more likely) on the boundary of the Kirtland lower shale (Hunter Wash Member) and overlying Farmington Sandstone Member using the USGS 24k surficial geology map (Brown, 1982). Thus NMMNH P-50000 occurs stratigraphically between *Terminocavus* holotype NMMNH P-27468, and the De-na-zin Member chasmosaurine fragment NMMNH P-33906. As such, NMMNH P-50000 is the stratigraphically highest reasonably complete skull known from the Campanian of the San Juan Basin.

**Morphology**

Because NMMNH P-50000 occurs high in the Kirtland Formation, then it might be expected to exhibit morphology intermediate between *Pentaceratops* and *Anchiceratops,* rather than simply being the same as *Pentaceratops*. Some features seem to support this: notably, the nasal horn is positioned more anteriorly than in *Pentaceratops*, and the narial strut of the premaxilla is oriented closer to vertical than the anteriorly inclined condition seen in c.f. *Pentaceratops* (see character matrix of Sampson et al., 2010), although this character may be influenced by ontogenetic elongation of the rostrum. Further information regarding morphology will be available upon completion of the description by J. Fry or S.G. Lucas.

## Taxa similar to Pentaceratops, from outside the San Juan Basin

### aff. *Pentaceratops* *sp.*; SDMNH 43470

Diem and Archibald (2005) described SDMNH 43470, a partial skull (including the midline of the parietal posterior bar) of a chasmosaurine from the Williams Fork Formation, Colorado. Despite similarities with *Pentaceratops*, Diem and Archibald (2005) refrained from referring the specimen to a specific genus. Later, Lucas et al. (2006) assigned SDMNH 43470 to *Pentaceratops sternbergii*, whereas Longrich (2014) suggested it may belong to the newly named taxon *P. aquilonius* (see later).

**Stratigraphy**

Diem and Archibald (2005) illustrate SDMNH 43470 as having been collected from the lower part of the Williams Fork Formation (Piceance basin, Rio Blanco County, Colorado), although no explanation is given in the text other than a note in the figure caption that this stratigraphic position is approximate (consultation of Diem's 1999 MS thesis reveals no additional data). Age of the Williams Fork Formation is constrained by the ammonites *Exiteloceras* *jenneyi* (75.08–74.60 Ma; Ogg and Hinnov, 2012) at the top of the underlying Iles Formation; *Didymoceras* *cheyennense* (74.60–74.21 Ma; Ogg and Hinnov, 2012), in the lower part of the Williams Fork Formation; and *Baculites reesidei* (73.63–73.27 Ma; Ogg and Hinnov, 2012) from a marine shale (below the Twenty Mile Sandstone Member) in the upper part of the Williams Fork Formation (Newman, 1987). Brownfield and Johnson (2008) published a K-Ar date of 72.5 ± 5.1 Ma from the Yampa bed tonstein which occurs 0–80 m above the base of the Williams Fork Formation (ie. very low) in more eastern exposures. For more discussion on age of the Williams Fork Formation, see Fowler (2017). From this data, it is likely that SDMNH 43470 derives from the *D. cheyennense* zone (74.60–74.21 Ma; Ogg and Hinnov, 2012), which is correlative with the upper half of the Hunter Wash Member (~75–74 Ma; Fowler, 2017) of the Kirtland Formation, in the San Juan Basin, New Mexico. However, the morphology of the specimen is more similar to chasmosaurines from stratigraphically lower (see below).

**Morphology**

Description and comparisons of SDMNH 43470 were given by Diem and Archibald (2005), and Lucas et al. (2006), so discussion here will be brief. The specimen was described as a subadult based on frill texture, a squamosal that is less elongate than other chasmosaurines, and visible cranial sutures (especially at the base of frill epiossifications). To this we can add the shape of the frill epiossifications, which are quite pointed, suggesting a relative maturity between juvenile and subadult ("adult" epiossifications are more broad and blunt; based on *Triceratops*; Horner and Goodwin, 2008). Despite its immaturity, the medial portion of the parietal is comparable in size to specimens of *Pentaceratops* and related chasmosaurines (Fig. 7). However, the squamosal is not as elongate as in (for example) aff. *Pentaceratops* n. sp. MNA PL.1747; this pattern is similarly seen in *Chasmosaurus russelli* specimen TMP 1983.25.1, which also has a large parietal of comparable size to (probable) adult *Chasmosaurus* specimens, but exhibits more pointed epiparietals, and a much shorter and broader squamosal. The presence of a flange protruding into the parietal fenestra from the ventral part of the posterior bar probably indicates that the parietal fenestrae are still opening; in all neoceratopsians with documented growth series, parietal fenestrae open and enlarge through ontogeny (e.g. Brown et al., 2009; Scannella and Horner, 2010; Fastovsky et al., 2011).

Parietal morphology of SDMNH 43470 is most similar to cf. *Pentaceratops* *sternbergii* material from the Fruitland Formation, and older chasmosaurines such as *Utahceratops* (Sampson et al., 2010). The median bar has a rounded cross section; the anteroposterior thickness of the (albeit incomplete) posterior bar is relatively uniform and strap-like, most similar to *Utahceratops* specimen UMNH VP-16671; the angle formed by the lateral rami of the posterior bar is 98°, slightly shallower than Fruitland Formation c.f *Pentaceratops* (83–88°) but much shallower than either of the new taxa (*Navajoceratops*, SMP VP1500, 60°; *Terminocavus* NMMNH P-27468, 73°). It is worth noting that Longrich (2014; p. 302) states that SDMNH 43470 "lacks the strongly U-shaped parietal emargination seen in [*Utahceratops gettyi* and *P. sternbergii*]". However, SDMNH 43470 clearly exhibits a U-shaped embayment (emargination), comparable to *Utahceratops* and cf. *P. sternbergii* (Fig. 7). What Longrich (2014, p. 297) actually refers to here is that in *Utahceratops* and *Pentaceratops* "the posterior margin of the parietal actually extends forward ahead of the end of the parietal fenestrae"; which is here interpreted as meaning that the deepest (anteriormost) point of the parietal emargination (which is positioned at the midline on the posterior edge of the posterior bar) is more anterior than the posteriormost edge of the parietal fenestrae (as measured on the anterior edge of the posterior bar). It is possible that this is exhibited by SDMNH 43470, although the posterior extent of the parietal fenestrae is not known as the lateral rami are not preserved more laterally than locus ep2. Regardless, the significance of this feature is difficult to assess as it is not observed in any other specimens of cf. or aff. *Pentaceratops* (contra. Longrich, 2014; see entry on *P. aquilonius*), and might be affected by ontogeny. However, the anterior depth of the median embayment is particularly deep in *Navajoceratops* (more so than in any cf. or aff. *Pentaceratops*) and development of this feature may be important in the eventual enclosure of the median embayment (as seen in *Terminocavus* through *Anchiceratops*; see main text). The position of the attached right ep2 may give indication as to the shape of the lateralmost parts of the lateral rami of the posterior bar. The anteriormost edge of the right lateralmost corner of the right ep2 must lie on the posterior edge of the right lateral ramus of the posterior bar. In which case, the posterior bar cannot have continued laterally at the same angle as seen in specimens referred to aff. *P*. n. sp. (MNA Pl.1747 and KUVP 161000). Instead, this morphology suggests that the posterior bar must have been angled at the ep2 locus, as seen in *Utahceratops* and cf. *P. sternbergii* (AMNH 1625). This might not be indicative of phylogenetic similarity with *Utahceratops* and cf. *P. sternbergii* as immature dinosaurs often exhibit character states more similar to the ancestral form than to an adult of their own species. However, it is a subtle but useful feature which aids in interpretation of the specimen.

The significance of the morphological similarity of SDMNH 43470 with Fruitland Formation (and more basal) chasmosaurines is twofold. Firstly, as currently understood, the stratigraphic position of the specimen (*D. cheyennense* zone; 74.60–74.21 Ma; Ogg and Hinnov, 2012) correlates with the upper half of the Hunter Wash Member (~75–74 Ma; Fowler, 2017) of the Kirtland Formation; i.e. stratigraphically equivalent to *Terminocavus*, and therefore higher than the Fruitland Formation (and older) chasmosaurines with which it is most similar morphologically. This is unexpected and may be indicative that the correlation of either or both units is incorrect; given the tentative nature of the Williams Fork stratigraphy, and the age of the analyses that produced the San Juan Basin radiometric dates, this seems likely. Secondly, the morphology of SDMNH 43470 may be reflective of its relative immaturity; young dinosaurs often appear more basal than adults of the same species (Fowler et al., 2011; Rozhdestvensky 1965). However, we do not think that this is the case, as the overall stratigraphic and phylogenetic trend in chasmosaurine parietal morphology is paedomorphism. Therefore, SDMNH 43470 cannot be confidently assigned to *Pentaceratops sternbergii* (*sensu* Lucas et al., 2006) nor *P. aquilonius* (Longrich, 2014; possibly a nomen dubium, see below). Instead, here a more conservative approach is suggested in considering it as aff. *Pentaceratops* sp., consistent with the original description (Diem and Archibald, 2005). Discovery of immature chasmosaurines from the Fruitland and Kirtland Formations, or more mature material from the Williams Fork Formation, may help clear up the issue.

### *Utahceratops gettyi*

Sampson et al. (2010) described the new taxon *Utahceratops gettyi* based on material from seven localities (including two multi individual bonebeds), spread over ~0.5–1 myr, of the lower to middle Kaiparowits Formation (Sampson et al., 2010; Loewen et al., 2013).

There are potential complications in that some of the characters that diagnose *Utahceratops gettyi* are not observable in all referred specimens. It is not explicitly stated which elements overlap among specimens (nor is there a list of elements which are present in the holotype UMNH 16784), making it unclear what justification there is for assigning each specimen to *Utahceratops gettyi*. Most critical here is the character "supraorbital horncores short", which is observed in the fragmentary skull UMNH 12198, but is not figured as occurring in the holotype. Loewen et al. (2013) state that an "orbit" is known for the holotype, but do not figure the specimen. For the time being, here we are going to follow the diagnosis of Sampson et al. (2010), but this will need to be reviewed in the future.

Two specimens preserve the middle part of the parietal posterior bar and are thus relevant to this study; holotype UMNH VP 16784 and UMNH VP 16671. These specimens are comparable in morphology (see below) and so for the purposes of this study they will be considered as representative of *Utahceratops*.

**Stratigraphy**

The stratigraphic position of specimens referred to *U. gettyi* were plotted by Loewen et al. (2013) relative to a series of radiometrically dated bentonites, the precise positions and ages of which are most recently summarized in Roberts et al. (2013). The stratigraphic position of the holotype specimen (UMNH VP 16784, locality UMNH 960) is shown by Roberts et al. (2013) as occurring in the lower part of the middle unit of the Kaiparowits Formation, slightly above an ash dated at 75.97 Ma (the distance in meters is unclear); however, what is presumably the same locality (mislabeled as "UMNH VP 16748") is shown by Loewen et al. (2013) as occurring ~175m above the base of the Kaiparowits Formation, slightly below the same radiometric date. The complete posterior bar, UMNH VP 16671, derives from a bonebed (locality UMNH 942; Sampson et al., 2010) that is ~105m higher than the holotype in the middle Kaiparowits Formation, ~280m above the base, and ~140 m below an ash horizon dated at 75.51 Ma (Loewen et al., 2013; Roberts et al., 2013). A conservative view (based on parietal morphology) of the stratigraphic range of *U. gettyi* is therefore ~75.97 Ma to ~75.6 Ma.

**Morphology**

Partial skull UMNH VP 16784 includes a near complete right ramus of the parietal posterior bar. UMNH VP 16671 is an isolated parietal, but is more complete, comprising a nearly complete posterior bar. Neither specimen preserves the lateralmost edge of the posterior bar where it would articulate with the squamosal. Both UMNH VP 16784 and 16671 exhibit a central embayment medial to the ep2 locus, which in both specimens extends anteriorly beyond the posteriormost border of the fenestrae. Only UMNH 16671 preserves both lateral rami, which meet at an angle of 75°. This is relatively acute, more so than in specimens of c.f. *Pentaceratops sternbergii* (83–88°). The posterior bar is of similar anteroposterior thickness from medial to lateral. However, this varies somewhat, with the right side of UMNH VP 16671 being narrower at the point between ep1 and ep2, and the left side being narrower at the lateralmost preserved point (at ep3). In both specimens, three epiparietals are preserved fused to each lateral ramus of the posterior bar, although the right ramus of UMNH VP 16671 is broken before the ep3 locus. Ep1 is an elongate dorsally-deflected ridge that extends along the medial edges of the embayment. Ep2 is triangular in shape, and projects posteriorly from the posterior bar which forms an obtuse angle at the ep2 locus, similar to that seen in *Pentaceratops sternbergii* specimen AMNH 1625. Ep3 is triangular in shape and projects posterolaterally. The median bar is incomplete and it is not possible to confidently determine the point of maximum constriction. Narrow lateral flanges extend from the median bar into the parietal fenestrae. These are similar in extent to *Navajoceratops*, but less developed than Taxon C. Although a fragment of a parietal lateral bar is preserved in UMNH VP 12198, it is not complete enough to tell whether the lateral bars completely enclosed the fenestrae.

### *Pentaceratops aquilonius*

Longrich (2014) recently named a new species of *Pentaceratops, P. aquilonius*, based on two specimens (holotype CMN 9813; paratype CMN 9814) from the uppermost Dinosaur Park Formation, Alberta, and possible referred specimen (SDMNH 43470) from the Williams Fork Formation, Colorado.

Holotype CMN 9813 comprises the distal end of a squamosal (bearing four episquamosals) and lateralmost part of the posterior bar of the parietal (bearing two fused epiparietals, probably ep3, and ep2). Paratype CMN 9814 comprises one fragment of the right lateral ramus of the posterior parietal bar, with two fused epiparietals (probably ep1 and ep2), and two isolated epiossifications labeled as ep1 and ep2 of the left side (Longrich, 2014). CMN 9813 and CMN 9814 were originally described by Langston (1959) and referred to *Anchiceratops* based on the large triangular epiparietals. SDMNH 43470 is a partial skull (including the midline of the parietal posterior bar) originally described by Diem and Archibald (2005) and referred to Chasmosaurinae sp., then to *P. sternbergii* by Lucas et al. (2006), and is here considered as aff. *Pentaceratops*.

Here it is shown that the autapomorphies used to diagnose *P. aquilonius* are invalid, and it should be considered a nomen dubium.

**Stratigraphy**

CMN 9813 and CMN 9814 were collected at different localities that expose the uppermost Dinosaur Park Formation, near Manyberries in southeast Alberta; i.e. outside of the well studied exposures in Dinosaur Provincial Park itself (details provided in Langston, 1959; and Longrich, 2014). Langston (1959) described the CMN 9813 locality as being "but a few feet" stratigraphically below an outlier of the Bearpaw Shale that conformably overlies the upper part of the Dinosaur Park Formation (then referred to as an undivided Oldman Formation), and described CMN 9814 as having been collected one meter below the Bearpaw Shale. He therefore equated both localities as occurring within the Lethbridge Coal Zone, which occurs at the top of the Dinosaur Park Formation. CMN 9813 and 9814 are thus most likely constrained in age by two radiometric dates; the base of the Lethbridge Coal Zone (LCZ Tuff; 76.10 ± 0.5 Ma; Eberth, 2011) and the base of the Bearpaw Shale (Bearpaw Tuff; 75.46 ± 0.24 Ma; Eberth, 2011). This overlaps with the range of the morphologically similar *Utahceratops gettyi* from the middle unit of the Kaiparowits Formation, Utah (~75.97 Ma to ~75.6 Ma ; see above; Sampson et al., 2010; Loewen et al., 2013; Roberts et al., 2013), which, along with *P. sternbergii*, was recovered as the sister taxon to *P. aqulilonius* by Longrich (2014). However, it is possible that the Lethbridge Coal Zone at Manyberries is slightly older than in Dinosaur Provincial Park (from which the radiometric dates are derived), since the coal zone tracks the shoreline of the westerly transgressing Bearpaw Seaway (Eberth, 2005), and Manyberries is ~80 km east of Dinosaur Provincial Park (and ~150 km east of Lethbridge).

**Morphology**

Longrich (2014) diagnosed *P. aquilonius* based on four autapomorphies, which are shown here as invalid, rendering *P. aquilonius* a nomen dubium:

1. Weak emargination [embayment] of parietal, with lateral rami meeting at an angle of approximately 90° and U-shaped emargination [embayment] not strongly extended anteriorly.

This autapomorphy cannot be assessed on *P. aquilonius* holotype CMN 9813 as it does not preserve the medial part of the posterior bar. Although the provided reconstruction (Longrich, 2014; fig. 6) suggests that enough of the posterior bar is preserved in CMN 9813 to accurately reconstruct the embayment, when shown to the same scale as AMNH 1625 (Fig. S17) it can be seen that the reconstruction probably overestimates the mediolateral extent of the posterior bar that is preserved in CMN 9813. Paratype CMN 9814 preserves the right lateralmost edge of the embayment, but it is not possible to assert its shape. Neither CMN 9813 nor 9814 preserve both lateral rami so the angle at which the rami meet is not measurable. None of these features can therefore be reliably observed in either the holotype or paratype.

2. [E]p1 projects dorsally, rather than anteriorly.

This autapomorphy cannot be assessed on the *P. aquilonius* holotype (CMN 9813) as it does not preserve the ep1 epiossification, or an ep1 locus (Langston, 1959; Longrich, 2014). For referred specimen CMN 9814, the isolated epiossification labeled as the left ep1 (Longrich, 2014) is unattached to the parietal and so cannot be assessed for orientation. The small preserved fragment of the right posterior bar of CMN 9814 exhibits ep1 and ep2 fused to the posterior margin and indeed, ep1 projects dorsally, rather than anteriorly. However, this character is variable in specimens of cf. *P. sternbergii*. The orientation of ep1 in CMN 9814 is compared (Longrich, 2014) with cf. *P. sternbergii* specimen AMNH 1625, which preserves left and right ep1 in different orientations: the left ep1 projects anterolaterally, whereas the right ep1 is almost identical to CMN 9814 in projecting dorsally. It is suggested that the dorsal projection of the right ep1 of AMNH 1625 is a preservational artifact, whereas that of the left ep1 is natural (Longrich, 2014). It is hard to imagine a burial process where the left ep1 remains as normal, whereas the right side is distorted to deflect dorsally, especially in this case where there is otherwise no unusual distortion elsewhere on the frill. Furthermore, it would seem easier if not more likely for a burial process to force down a dorsally projected epiossification to make it anteriorly projecting, rather than raising an anteriorly projecting epiossification into a dorsally projecting orientation. This character is therefore shown to be individually and/or taphonomically variable and not taxonomically informative.

3. Lateral epoccipitals [episquamosals] <100 mm in length.

This character refers to the length of the bases of the four episquamosals present on CMN 9813. It has been shown in *Triceratops* that the shape of frill epiossifications changes through ontogeny; in young animals the epiossifications are narrow-based and more pointed, which then broaden at the base and become blunt through to adulthood (Horner and Goodwin 2006; 2008; Scannella and Horner, 2010). It is possible, therefore, that the small size of the episquamosals in CMN 9813 is because the specimen is not fully mature. Morphology of the episquamosals is slightly pointed (more so than in AMNH 1625), which supports the hypothesis that CMN 9813 is relatively less mature than AMNH 1625. Furthermore, although episquamosal size follows general patterns based on position (ie. the posteriormost episquamosal is usually the largest, seen in CMN 9813), absolute size of lateral episquamosals is variable, even within individuals (e.g. CMN 2280), such that this is probably not a very reliable character upon which to base a species diagnosis.

4. Caudal [posterior] bar of parietal anteroposteriorly broad relative to *P. sternbergii* or *Utahceratops gettyi*.

This character can only be assessed in holotype CMN 9813 as the paratype CMN 9814 is too incomplete. Longrich (2014) reconstructs CMN 9813 with a posterior bar that appears to be anteroposteriorly thicker than cf. *P. sternbergii* AMNH 1625, to which it is being compared. However, this is at least partly an artifact of scaling issues between the specimens. Figure S17 shows both CMN 9813 and AMNH 1625 at the same scale, whereupon it is clear that the posterior bar of CMN 9813 is comparable in anteroposterior thickness to AMNH 1625 (and other specimens of cf. *P. sternbergii*; see Fig. 7). Furthermore, in all neoceratopsians with documented growth series, the parietal fenestrae open and enlarge through ontogeny by resorption of bone at the margins of the fenestra (e.g. Brown et al., 2009; Scannella and Horner, 2010; Fastovsky et al., 2011). If CMN 9813 is not yet fully mature, then the fenestrae might be enlarged by further resorption of the anterior margin of the posterior bar.

In conclusion, here it is agreed with Longrich (2014) that CMN 9813 and 9814 are morphologically more similar to material referred to *Utahceratops* *gettyi* and cf. *Pentaceratops sternbergii*, than to *Anchiceratops ornatus* (as suggested by Langston, 1959), and probably represent a northern representative of the *Utahceratops* - *Pentaceratops* clade. However, the type and referred specimens are extremely fragmentary, making a specific diagnosis difficult; the autapomorphies used to diagnose *P. aquilonius* are here shown to be invalid, and it should be considered a nomen dubium. Given that *P. aquilonius* overlaps stratigraphically with *Utahceratops gettyi*, then it seems likely that the two taxa might be very closely related, if not conspecific. However, it is not possible to assert this based on currently published material.

### *Bravoceratops polyphemus*

Wick and Lehman (2013) recently described a partial chasmosaurine skull (TMM 46015-1) from the lowermost Javelina Formation, Texas, as the holotype of *Bravoceratops polyphemus*. The characters that diagnose *B. polyphemus* almost all occur on the parietal posterior bar and median bar: described as fan-shaped, lacking a median embayment, and exhibiting a raised midline area with a shallow depression suggested to represent a missing midline epiparietal (Wick and Lehman, 2013). Although we sympathize with the overall conclusions of this paper, the median bar is misidentified, and as such none of the characters used to diagnose *B.* *polyphemus* are actually present in the material.

**Morphology & reassessment**

The element identified as the posterior half of the median bar of the parietal is almost certainly the anterior half (Fig. S16), comparing favorably with anterior median bars of other chasmosaurines, including well-figured specimens USNM 8604 (c.f. *Pentaceratops*; Kirtland Formation, New Mexico; Gilmore, 1919); PMU R223 (c.f. *Pentaceratops*; Kirtland Formation, New Mexico; Wiman, 1930; Fig. 1, no.s 5, 6); CMN 491 (the holotype of *Chasmosaurus belli*; as illustrated in Hatcher et al., 1907; Pl. XXI), and NHMUK R4948 (*C. belli*; Maidment and Barrett, 2011). In these specimens the median bar expands laterally at its anterior end, and on the ventral side hosts a shallow rounded fossa, bordered by distinctive longitudinal grooves typical of the ventral side of chasmosaurine parietals (most obvious in Hatcher et al., 1907, Pl. XXI; well-described by Maidment and Barrett, 2011). This matches the morphology of TMM 46015-1 if the element is rotated 180 degrees anteroposteriorly. By contrast, the midpoint and posterior end of chasmosaurine median bars are flat to convex ventrally (i.e. unlike the described condition of TMM 46015-1 in the incorrect orientation). Furthermore, a raised midline ridge on the posterior end of the median bar (as proposed for *Bravoceratops* Wick & Lehman, 2013) is not observed in other chasmosaurines, but a ridge is commonly observed on the anterior end (e.g. *Anchiceratops*; Brown, 1914; Mallon et al., 2011; "*Torosaurus*" *utahensis*; Gilmore, 1946; Lawson, 1976; Sullivan et al., 2005; c.f. *Pentaceratops*; Gilmore, 1919; "*Titanoceratops*" c.f. *Pentaceratops*; Longrich, 2011; *Arrhinoceratops*; Mallon et al., 2014; and subtly in *Chasmosaurus belli*; Hatcher et al., 1907; Maidment and Barrett, 2011). A series of raised bumps along the midline of the parietal is typical of immature *Triceratops* ("raised bony prominences"; Goodwin et al., 2006), which become less pronounced through to adulthood, and are also seen *Coahuilaceratops* (Loewen et al., 2010; Cerro del Pueblo Formation, Mexico), which is closely related to, and possibly contemporaneous with *Bravoceratops*. We therefore suggest here that the pit present on the midline of TMM 46015-1 does not represent attachment of an epiparietal, rather, it is a posterior midline bump in the process of being remodeled (a similar depression on a raised midline is visible on the dorsal surface on USNM 8604; Gilmore, 1919; see Fig. S16). Contrary to the reconstruction given by Wick and Lehman (2013; figure 6f; see Fig. S16), there does not appear to be any indication of an epiparietal present on what is described as the posterior margin of the parietal. Finally, a bifurcated quadratojugal (articulation surface of the squamosal) is given as an autapomorphy of *Bravoceratops*, allying it to "*Torosaurus*" and *Triceratops* (Wick and Lehman, 2013). However, a bifurcated quadratojugal-squamosal articulation surface is not visible in any of the *Triceratops* specimens held at MOR examined by D. Fowler and J. Scannella; the bifurcation seen in the illustrated examples is probably the result of the squamosal overlapping the quadratojugal, and might not be a genuine anatomical feature of *Bravoceratops*. Although *Bravoceratops polyphemus* should therefore be considered a *nomen dubium*, TMM 465015-1 remains important as it is morphologically most similar to chasmosaurines from the late Campanian.

Re-identification of the parietal fragment means that there is relatively little remaining diagnostic morphology of TMM 46015-1 for comparison. However, the preserved portion of the parietal shows that the median bar is relatively narrow, suggesting that quite large parietal fenestrae were present. These large parietal fenestrae are unlike the reduced fenestrae seen in derived chasmosaurines from the Maastrichtian (*Anchiceratops*, *Arrhinoceratops*, and Triceratopsini) or that of Campanian chasmosaurines *Vagaceratops* and *Kosmoceratops*. The lack of lateral flanges to the median bar is also unlike Kirtland Formation Taxon C (NMMNH P-33906). This suggests that TMM 46015-1 is a chasmosaurine less derived than Kirtland Taxon C, and therefore probably older than the De-na-zin Member of the Kirtland Formation (73.8–73.5 Ma; Fowler, 2017; see below).

**Stratigraphy**

The age of TMM 46015-1 remains uncertain, and can only be constrained to between the middle Campanian to early Maastrichtian by bounding radiometric dates and/or ammonite biostratigraphy. Wick and Lehman (2013) state that TMM 465015-1 derives from the lowermost Javelina Formation, ~3 m above the contact with the underlying Upper Shale Member of the Aguja Formation, and therefore suggest that the specimen is likely to be from the late Campanian to early Maastrichtian. Although we agree with this interpretation, the age of the Aguja and Javelina formations are imprecisely known, and might be of longer (or more intermittent) duration than is typically shown (Fowler, 2017). The Upper Shale Member of the Aguja Formation rests upon the Terlingua Creek Sandstone, which is dated by the presence of the ammonite *Baculites mclearni* (Rowe et al., 1992; 80.67–80.21 Ma; lowermost part of the middle Campanian; Ogg & Hinnov, 2012). Two U-Pb radiometric dates related to phreatomagmatic volcanism have been published as from within the Upper Shale Member of the Aguja Formation; 76.9 +/- 1.2 Ma (Befus et al., 2008), and 72.6 +/- 1.5 Ma (Breyer et al., 2007). As the only radiometric dates reported from the Aguja Formation, it seems reasonable to report these dates as representing the age of the Upper Shale Member. However, Befus et al. (2008) present a model showing that two explosive volcanic events created craters within what would traditionally be considered as the Upper Shale Member; i.e. that the volcanism occurred after the Upper Shale Member had been deposited. As such, these dates might not in fact be representative of the age of the Upper Shale Member of the Aguja Formation. Elsewhere in the Big Bend region of southern Texas, the Javelina Formation overlies the Upper Shale Member of the Aguja Formation. Although the Javelina Formation is often cited as being ~69 Ma in age (due to a U-Pb date of 69 +/- 0.9 reported by Lehman et al., 2006), it is entirely possible that the base of the Javelina is older as the 69 Ma datum occurs approximately mid-way through the Javelina Formation. Thus the age of TMM 46015-1 can really only be constrained as between the top of the *B. mclearni* zone (80.21 Ma) to the 69 Ma datum.

Based on the revised morphology of *Bravoceratops* (see above), we suspect that TMM 46015-1 is probably from the late Campanian, older than Kirtland Formation Taxon C (De-na-zin Member, 73.8–73.5 Ma; Fowler, 2017). This is important as it would suggest that the basal part of the Javelina is older than previously assumed and might bear a fauna comparable to similarly aged units in Utah and New Mexico, potentially adding new data to the debate surrounding northern and southern biogeography. This hypothesis is potentially supported by the recent description of *Kritosaurus* sp. material from the lowermost Javelina Formation (Lehman et al., 2016), a genus of hadrosaurid dinosaur otherwise known only from the late Campanian.

## Chasmosaurus and related chasmosaurines from the Western Interior

*Chasmosaurus* is a genus of ceratopsid dinosaur from the middle to upper Campanian Dinosaur Park Formation of Alberta, Canada (and Aguja Formation, Texas). It is important as it is one of the oldest well-known representatives of the Chasmosaurinae. *Chasmosaurus* has an incredibly complex taxonomic history (especially recently) due to synonymization of many historical taxa (*C. belli*; *C. brevirostris*; *Eoceratops canadensis*/ *C. canadensis*; *C. kaiseni*; *C. russelli*), followed by the erection of many new taxa, some of which are based on historical specimens (*C. mariscalensis*; *C. irvinensis*/ *Vagaceratops irvinensis*; *Mojoceratops perifania*; *Kosmoceratops richardsoni*; *Kosmoceratops* sp.). A detailed review of the taxonomic history of *Chasmosaurus* can be found in Godfrey and Holmes, 1995, and Maidment and Barrett, 2011. This taxonomic complexity is a result of multiple fragmentary specimens combined with morphological variability which, as with other dinosaurs, is probably strongly influenced by differences in stratigraphic position and ontogenetic age. The stratigraphic distribution of *Chasmosaurus* (and closely related taxa) is relatively well understood, although complicated by the complex taxonomic history and some inconsistency regarding the stratigraphic placement of a critical specimen (YPM 2016; see below, and main text). A brief treatment of ontogenetic change in *Chasmosaurus* was discussed by Lehman (1989; 2007) and greatly expanded by Campbell et al. (2016).

Godfrey and Holmes (1995) reviewed specimens assigned to *Chasmosaurus* and reduced the genus to two valid species; *C. belli* and *C. russelli*, based on the morphology of the posterior bar of the parietal. Later, Holmes et al. (2001) described a third species, *C. irvinensis*, which was renamed as *Vagaceratops* *irvinensis* by Sampson et al. (2010) and is therefore discussed later in this document. Campbell et al. (2016) review the genus, preserving *C. russelli* and *C. belli*. To begin with we will review the recently named taxon *Mojoceratops perifania* (Longrich, 2010).

### "*Mojoceratops perifania*"

Longrich (2010) recently described the chasmosaurine ceratopsid *Mojoceratops perifania* based on eight cranial specimens (holotype, TMP 1983.25.1; referred specimens, TMP 1999.55.292; NMC 8803; AMNH 5656; AMNH 5401; CMN 1254; CMN 34893; TMP 1979.11.147) previously assigned to *Chasmosaurus russelli*, *C. belli*, *C. kaiseni* (considered a junior synonym of *C. belli*) and *C.* (*Eoceratops*) *canadensis* (also considered a junior synonym of *C. belli*). This has invoked a discussion over the validity of *M. perifania*, with complex issues surrounding taxonomic priority, morphological change through ontogeny, and stratigraphic consistency, among others.

**Taxonomy**

Two taxonomic issues concern *Mojoceratops perifania*. Firstly, Longrich (2010) refers the nearly complete skull AMNH 5401 and partial skull CMN 1254 to *M. perifania*; However, since AMNH 5401 was originally described as the holotype of *Chasmosaurus kaiseni* (Brown, 1933), and CMN 1254 was described as the holotype of *Eoceratops canadensis* (Lambe 1902; 1915; later *C. canadensis*; Lehman, 1990) then if either specimen is indeed the same taxon as the *M. perifania* holotype (TMP 1983.25.1) at the least the new taxon should have become some taxonomic combination of *E. canadensis* and *C. kaiseni* (probably *E. canadensis* has priority). Secondly, the validity of *M. perifania* itself was questioned by both Mallon et al. (2011), Maidment and Barrett (2011), and Campbell et al. (2016), who considered it a junior synonym of *C. russelli*, thereby potentially avoiding the aforementioned taxonomic issue. Indeed the holotype of *M. perifania* resembles closely a referred specimen of *C. russelli* (NMC 2280; see later). However, this proposed synonymy is complicated by the recent finding that the holotype of *C. russelli* may not be the same taxon as the referred specimens to which *M. perifania* is compared (see later).

**Stratigraphy**

Of the eight specimens referred by Longrich (2010) to *M. perifania*, only TMP 1979.11.147 can be placed stratigraphically with any precision, and this is of little use as the specimen comprises only a set of brow horns that should probably be considered as Chasmosaurinae sp. (Godfrey and Holmes, 1995). NMC 8803 was collected in Saskatchewan from a locality thought to be equivalent to the middle part of the Dinosaur Park Formation. All other specimens have either only general provenance data, or lack locality data altogether. However, the holotype of *M. perifania* (TMP 1983.25.1) compares most favorably with specimens previously attributed to *C. russelli* from the lowermost part of the Dinosaur Park Formation, Alberta, and was considered a junior synonym of *C. russelli* by Maidment and Barrett (2011) and Mallon et al. (2011).

As stated below, the holotype of *C. russelli* is from the uppermost part of the Dinosaur Park Formation; this is much higher in section than all the referred specimens, which derive from the lower part of the Dinosaur Park Formation. If these lower specimens are eventually considered a different taxon from the holotype of *C. russelli,* and from *C. belli*, then either *M. perifania*, *C. kaiseni, C. priscus,* or *E. canadensis* (or some combination thereof) might become valid.

**Morphology and ontogeny**

The holotype (TMP 1983.25.1) and at least one of the referred specimens (AMNH 5656, a small but complete frill and basicranium) of *Mojoceratops perifania* are probably from young individuals (small size, lack of cranial fusion, pointed and/or unfused frill epiossifications; crenulated frill margin; squamosal only weakly elongated; lack of resorption of postorbital horns; Lehman, 1990; Ryan and Evans, 2005; Horner and Goodwin, 2006; 2008; Scannella and Horner, 2010; Campbell et al., 2016). As such, their morphology might reflect an immature status rather than their "true" adult state, and similarities between specimens may be due to a similar ontogenetic stage rather than truly representing the same taxon. This is especially important for AMNH 5656 as its stratigraphic and geographic provenance is unknown (Tanke, 2010); although it is similar in morphology to TMP 1983.25.1 (referred to *C. russelli* by Maidment et al., 2011 and Mallon et al., 2012), it is nearly half the size and could instead represent an immature individual from a stratigraphically higher taxon such as *C. belli*.

Regardless of these many issues, here we follow Maidment et al. (2011) and Mallon et al. (2012) in considering *Mojoceratops perifania* a synonym of *Chasmosaurus russelli*.

### *Chasmosaurus belli, "Chasmosaurus russelli", and Chasmosaurus* sp.

**Taxonomy**

*Chasmosaurus belli* was originally described as *Monoclonius belli* by Lambe (1902) based on a relatively complete parietal (CMN 491), including the midline of the posterior bar. This was later placed into *Ceratops* by Hatcher (1907), then *Protorosaurus belli* by Lambe (1914a), but this was found to be preoccupied, so Lambe (1914b) changed it to *Chasmosaurus belli* while describing a new skull and skeleton (CMN 2245). Since this time many specimens have been referred to *C. belli* (see revisions by Lull, 1933; Godfrey and Holmes, 1995; Campbell et al., 2016; 2018; 2019). However, only six specimens have the diagnostic parietal posterior bar intact; holotype CMN 491 (Lambe, 1914a), CMN 2245 (Lambe, 1914b), AMNH 5402 (Lull, 1933); NHMUK R4948 (Maidment and Barrett, 2011); ROM 843 (Godfrey and Holmes, 1995), and YPM 2016 (Lull, 1933; note that although YPM 2016 is typically referred to *C. belli*, it exhibits morphology intermediate between more typical *C. belli*, and *Vagaceratops*). Campbell et al. (2019; p. 91) removed YPM 2016 and AMNH 5402 from *C. belli*, and along with new specimen TMP 2009.034.0009 (a fragment of a parietal posterior bar) instead refer to them as *Chasmosaurus* sp., suggesting they represent "stratigraphically-basal members of the *Vagaceratops irvinensis* clade" (this is discussed below).

*Chasmosaurus russelli* was described by Sternberg (1940) based on a very large complete skull (holotype CMN 8800), and referred specimens CMN 8801 (paratype; anterior part of a skull, no frill), CMN 8802 (partial skull without frill, destroyed in 1941; Tanke, 2010), and CMN 8803 (medial part of the parietal posterior bar). Godfrey and Holmes (1995) later referred the following specimens to *C. russelli* (some tentatively): AMNH 5656 (posterior half of a small skull with frill), CMN 2280 (large complete skull), TMP 1983.25.1 (posterior part of a skull, including frill), TMP 1981.19.175 (most of a skull, missing the posterior frill), and CMN 41933 (posterolateral corner of parietal). Longrich (2010) reassigned a number of specimens from *C. russelli* and *C. belli* into his new taxon, *Mojoceratops perifania*, which was considered invalid by Maidment and Barrett (2011; see section on *Mojoceratops*).

The taxonomy of *Chasmosaurus russelli* is currently in a state of flux as the holotype (CMN 8800) probably does not pertain to the same genus as most of the referred specimens (including the holotype of "*Mojoceratops perifania"* previously referred to *C. russelli*; Godfrey and Holmes, 1995; Longrich, 2010), indeed CMN 8800 may even be unrelated to the genus *Chasmosaurus* (Longrich, 2014; M. Loewen and D. Evans, pers. comm.; Fowler and Freedman Fowler, 2017). In reference to this issue, Longrich (2014) refers to *C. russelli* referred specimen CMN 2280 as *Chasmosaurus* n. sp. in his phylogenetic analysis, and does not include "*C. russelli*". We are avoiding any issues associated with this situation by referring to "*Chasmosaurus russelli*" specimens by specimen number (we also do not refer to the holotype specimen since it is poorly figured and rarely referred to by other researchers), thereby avoiding any confusion while we prepare publication of new research that resolves the matter (see Fowler and Freedman Fowler, 2017).

**Stratigraphy**

Stratigraphic positions of specimens (Table S1) are based on the topographic height of the localities given by Currie and Russell (2005). This data was reanalyzed by Mallon et al. (2012; supp. info.) to compensate for structural dip, and presented as the number of meters above the basal contact of the Dinosaur Park Formation with the underlying Oldman Formation. Mallon et al., (2012) state that there is an error of ±7 m for their stated measurements.

Precise stratigraphic position is only known for one specimen of "*C. russelli"* that exhibits the posterior parietal bar: CMN 2280 was recovered from 667 m above sea level (Currie and Russell, 2005), estimated at 15.9 m above the Oldman - Dinosaur Park Formation contact (Mallon et al., 2012), and therefore ~21.1 m below a bentonite dated at 76.39 Ma that occurs 36 m above the formational contact (dates from Eberth, 2011; see Fowler, 2017). Alternatively, Campbell et al. (2016) place CMN 2280 at ~7 m above the base of the Dinosaur Park Formation (this slight difference has no bearing on the discussion here). The holotype of *C. russelli* (CMN 8800) has more vague locality data, having been collected from high in the Dinosaur Park Formation from an area outside of Dinosaur Provincial Park itself (Sternberg, 1940). This was recently restudied with greater resolution by Campbell et al., (2013; 2016), whereupon it was found to have been collected high in the Dinosaur Park Formation (shown as ~54m above the formational base, ~1 m below the base of the Lethbridge Coal Zone). However as previously mentioned, CMN 8800 probably does not pertain to the same genus as many or all of the referred specimens (Longrich, 2014; M. Loewen and D. Evans, pers. comm., Fowler and Freedman Fowler, 2017). Of all other specimens referred to "*C. russelli"* only TMP 81.19.175 has locality data, which places it 659 m above sea level (Currie and Russell, 2005), estimated at 10.5 m above the Oldman - Dinosaur Park Formation contact (Mallon et al., 2012; Campbell et al., 2016).

Stratigraphic position is only known for five specimens of *C. belli* (or *C*. sp.; Campbell et al., 2019) that exhibit the posterior parietal bar (NHMUK R4948; ROM 843; CMN 2245, YPM 2016, and TMP 2009.034.0009), all of which derive from the middle to upper part of the Dinosaur Park Formation (given here in stratigraphic order), straddling a 76.39 Ma bentonite at 36 m above the Oldman - Dinosaur Park Formation contact, and below a 76.10 Ma bentonite at 61.5 m (both dates from Eberth, 2011; see Fowler, 2017). NHMUK R4948 was recovered from an estimated 20.1 m above the Oldman - Dinosaur Park Formation contact (Mallon et al., 2012; NHMUK R4948 is not mentioned by Currie and Russell 2005). ROM 843 was recovered from 682 m above sea level (Currie and Russell, 2005), estimated at 33.4 m above the Oldman - Dinosaur Park Formation contact (Mallon et al., 2012; Campbell et al., 2016). TMP 2009.034.0009 was recovered from ~34 m above the Oldman - Dinosaur Park Fromation contact (Campbell et al., 2019). Finally, CMN 2245 was recovered at 692 m above sea level (Currie and Russell, 2005), estimated at 41.0 m above the Oldman - Dinosaur Park Formation contact (Mallon et al., 2012; ~42 m in Campbell et al., 2016).

**YPM 2016**

Conflicting stratigraphic data has been published for YPM 2016. On the basis of field notes from C.M. Sternberg himself (see Longrich, 2015; Campbell et al., 2019), Lull (1933) recorded the locality of YPM 2016 as 60 feet [18.29 m] below the Pierre [Bearpaw] Shale. Campbell et al. (2019) report that Sternberg's own field notes (Sternberg, 1919) state more specifically "about 65 ft [20 m] below Pierre Shale on the south side of the west branch of Little Sand Hill Creek within about 3/4 of a mile from where the badlands give way to grass-covered slopes". A stratigraphic placement 20m below the Bearpaw (Pierre) shale would place the specimen in the uppermost part of the Dinosaur Park Formation, ~50–60 m above its base, and just below the Lethbridge Coal Zone which occurs at ~58 m above the formational base (see Longrich, 2015; Campbell et al., 2019). The more commonly reported stratigraphic position for YPM 2016 (e.g. in Currie and Russell, 2005; Mallon et al., 2012; Campbell et al., 2016; Campbell et al., 2019) is based on the results of a quarry staking exercise undertaken in 1935 and 1936 whereupon C.M. Sternberg and his son suggested that YPM 2016 was collected from quarry 110. The stratigraphic position of quarry 110 is 684 m above sea level (Currie and Russell, 2005), estimated at 24.5 m above the Oldman - Dinosaur Park Formation contact (Currie and Russell, 2005; Mallon et al., 2012; or 26.5 m in Campbell et al., 2016; 2019). It is important to note that the stratigraphic position of quarry 110 is ~20–30 m lower in section than suggested in C.M. Sternberg's 1919 field notes (see above).

Campbell et al. (2019) state that they consider the staked quarry 110 is indeed the source of YPM 2016, as quarry 110's location is consistent with the description given by Sternberg (1919). However, they would appear to mean that quarry 110's location is consistent only with the geographic description given by Sternberg (no detailed explanation is given), and it is important to recognise that their assertion of this contradicts Sternberg's original field observation that YPM 2016 came from 65ft below the Bearpaw Shale.

Longrich (2015) offers several possible and compelling reasons for the discrepancy between Sternberg's 1919 original field notes and the stratigraphic and geographic position of quarry 110. One suggestion was that it was the quarry staking excercise of 1935–36 that might be in error, i.e. that the original 1919 observation was correct and that quarry 110 was not the correct site for YPM 2016. Longrich (2015) supports this hypothesis by the fact that other quarries from the 1935–36 quarry staking exercise are known to have been misidentified (Tanke, 2005). Indeed, inconsistency between C.M. Sternberg's accounts of quarry locations are also reported from the archives of the Sternberg Museum, Hays, KS (D. Steffen, pers. comm.). Campbell et al. (2019) note that there is no exposure of the Bearpaw near to quarry 110, in which case quarry misidentification seems very plausible as it is hard to imagine why C. M. Sternberg would record in his field notes that the specimen was collected ~65ft below the Bearpaw shale if it was impossible to assert this: either by direct observation of the Bearpaw, or by measuring up from the basal contact of the Dinosaur Park Formation and making an estimate based on its typical thickness.

Here then we can only treat the quarry 110 location as dubious; this is of possible importance as YPM 2016 exhibits a morphology intermediate between typical *C. belli* and *Vagaceratops* (see later).

**CMN 2245**

A potentially important specimen in the light of the recent work of Campbell et al. (2016, 2019) is CMN 2245, the posterior half of a skull, including the diagnostic posterior bar of the parietal, which was originally assigned to *C. belli* (Lambe, 1914b). CMN 2245 was collected from relatively high in the Dinosaur Park Formation, ~41–44m above its base (see Table S1, below) which would make it the stratigraphically highest known specimen of *C. belli*, indeed of any *Chasmosaurus*, that preserves the parietal posterior bar. However, many features of CMN 2245 are recognised as indicative of immaturity (e.g. unfused epiparietals (other than ep3), weakly fused and pointed episquamosals, unresorbed postorbital horns, scalloped frill margin; see Campbell et al., 2016). Although this specimen is classified as "adult" in Campbell et al. (2016), of the 15 sampled specimens it is classified as the tenth least mature, and lacks 13 characters seen in the most mature specimens. This is significant as it is possible that CMN 2245 might represent an immature form of the same taxon as YPM 2016 and AMNH 5402, which were removed from *C. belli* by Campbell et al. (2019). Notably, CMN 2245 exhibits a large triangular epiparietal immediately adjacent to the parietal-squamosal contact, something otherwise only seen in specimens attributed to *C*. sp (Campbell et al., 2019). The taxonomic affinity of CMN 2245 does not appear to have been reconsidered by either Campbell et al. (2019; or any other analysis since), probably because it does not bear any indicaton of fused epiparietals other than in the lateralmost position. Nevertheless, there seems no longer any compelling reason to consider CMN 2245 as the same taxon as ROM 843. This may have important implications; however further study is required, beyond the scope of the current manuscript.

| Specimen | Taxon | Lull (1933) | Sternberg 1950 | Godfrey & Holmes 1995 | Currie & Russell 2005 | Mallon et al. 2012 | Campbell et al. 2016 |
| --- | --- | --- | --- | --- | --- | --- | --- |
|  |  |  |  |  |  |  |  |
|  |  |  |  |  |  |  |  |
|  |  |  |  |  |  |  |  |
| CMN 2245 | *C. belli* |  | 691.90 | 43.89 | x | 41.0 | ~42 |
| ROM 843 | *C. belli* |  | 681.78 | 35.77 | 682 | 33.4 | ~33 |
| YPM 2016 | *c.f. C. belli* | ~50–60 | 686.41 | 31.4 | 684 | 24.5 | ~27 |
| NHMUK R4948 | *C. belli* |  | 668.58 | N/A | N/A | 20.1 | N/A |
| CMN 8801 | *c.f. C. russelli* |  | 670.04 | 23.04 | x | N/A | N/A |
| CMN 2280 | *"C. russelli"* |  | 674.71 | 14.7 | 667 | 15.9 | ~7 |

Table S1. Stratigraphic heights of *Chasmosaurus* specimens which preserve the diagnostic posterior border of the parietal. Godfrey and Holmes (1995), Mallon et al. (2012), and Campbell et al. (2016) estimated the height in meters above the base of the Dinosaur Park Formation based on topographic maps. The Currie and Russell (2005) measurements are elevation in meters above modern sea level. Sternberg (1950) measurements are also in meters above sea level, converted from their original measurements in feet. Lull (1933) measurement for YPM 2016 converted from feet and based on a 60–70 m thick Dinosaur Park Formation (see Longrich, 2015).

*Chasmosaurus* has been presented as a good example of a stratigraphically separated morphospecies (Holmes et al., 2001); with *C. russelli* limited to the lower part of the Dinosaur Park Formation; *C. belli* the middle to upper part, and *C. irvinensis* (later referred to a new genus *Vagaceratops irvinensis*; Sampson et al., 2010; see later) restricted to the uppermost few meters (below the Lethbridge Coal Zone). The realization that the holotype of *C. russelli* is from the upper part of the Dinosaur Park Formation (Sternberg, 1940; Campbell et al., 2013; 2016) would change this, except that the holotype is suspected to be a different taxon from the referred specimens (see above). Therefore for this case the stratigraphic segregation of Holmes et al. (2001) is maintained. It should be noted that Mallon et al. (2012) show slight stratigraphic overlap between *C. russelli* and *C. belli*. This is based on the uppermost specimen referred to *C. russelli* (CMN 8801), which occurs at 670 m above sea level (Currie and Russell, 2005), estimated at 21 m above the Oldman - Dinosaur Park Formation contact (Mallon et al., 2012), and therefore overlaps with lowermost *C. belli* specimen NHMUK R4948. However, CMN 8801 comprises a well preserved anterior half of a skull, but lacks the frill and as such was not considered diagnostic to species level by Godfrey and Holmes (1995), who referred it only to *Chasmosaurus* sp. This conservative view therefore preserves the stratigraphic distinction of *C. russelli* and *C. belli*. Alternatively, CMN 8801 was assigned to *Kosmoceratops* sp. by Longrich (2014). Amid this disagreement, the taxonomic identity of CMN 8801 awaits detailed new work on Campanian chasmosaurines from Alberta.

Campbell et al. (2016; 2019) offer further discussion on the stratigraphic occurrence of various taxa within the *Chasmosaurus* clade, and contend that *C. belli* and *C*. sp show some stratigraphic overlap between ROM 843 and YPM 2016 (although stratigraphic placement of *Chasmosaurus* specimens is based on unconfirmed historical data with various recognized problems; also see above for uncertainty over the position of YPM 2016). Campbell et al. (2016; 2019) do not operate under the assumption here that *C. russelli* holotype CMN 8800 is not the same taxon as other specimens referred to *C. russelli,* nor the recognition that CMN245 might not belong to the same taxon as ROM 843 (this accounts for some stratigraphic overlap). Nevertheless, if real, stratigraphic overlap of YPM 2016 and ROM 843 might mean that an anagenetic lineage is not possible between *C. belli* and *C*. sp (although an ancestor-descendant relationship is still probable). This also makes assumptions about morphological variation within a population, something which is dependent upon large sample sizes which do not exist for most ceratopsids (with the possible exception of *Centrosaurus* and *Triceratops*.

**Morphology**

The morphology of *Chasmosaurus russelli* and *C. belli* is described by Godfrey and Holmes (1995) and Maidment and Barrett (2011) such that a detailed treatment is not required here. However, variation in the parietal among specimens warrants discussion, particularly with regards to phylogenetic and ontogenetic variability in characters considered important in *Pentaceratops* (and relatives), and a possible transitional specimen (YPM 2016) between typical *C. belli* and *Vagaceratops* (*Chasmosaurus*) *irvinensis*.

*"Chasmosaurus russelli"* referred specimens

CMN 2280 is the most complete of the specimens referred to "*Chasmosaurus russelli"* and is typically used as the reference for the morphology of the species (e.g. Godfrey and Holmes, 1995). However, the referred specimens vary slightly with regards to the shape of the posterior bar, the angle of the lateral rami, and the position of the epiparietals (Fig. S11). In all referred specimens the anteroposterior thickness of the posterior bar is either uniform mediolaterally, or tapers slightly from medial to lateral. The posterior bar is anteroposteriorly relatively narrow overall, similar to *Utahceratops*, but unlike that of more derived chasmosaurines where the posterior bar is more anteroposteriorly broad. In CMN 2280 the paired lateral rami of the posterior bar form a rounded M-shape, similarly seen in referred specimens AMNH 5656 and TMP 1983.25.1. Referred specimens TMP 1999.55.292 and CMN 8803 have M-shaped lateral rami that are more angular or subangular, similar to *Utahceratops*. In CMN 2280, AMNH 5656 and TMP 1983.25.1, the median embayment is formed from gradual medially inclined curvature of the lateral rami. By contrast, in CMN 8803 (and to a lesser extent, isolated right lateral ramus, TMP 1999.55.292) the median embayment is demarcated by a sharp change in angle of the lateral rami (more similar to *Utahceratops*, and especially the reconstruction of *Agujaceratops* provided by Lehman, 1989). The degree to which the median embayment extends anteriorly beyond the posteriormost extent of the parietal fenestrae is gradational and related to the angle of the lateral rami. In CMN 2280 the lateral rami of the posterior bar meet medially at 131°. By comparison the angle between the lateral rami is 99° in referred specimen AMNH 5656, 106° in TMP 1983.25.1, but only 87° in CMN 8803. Furthermore, in both CMN 8803 and TMP 1999.55.292, the anteriormost point of the median embayment extends only slightly beyond the posteriormost extent of the fenestrae in CMN 2280, extends moderately anteriorly in TMP 1983.25.1 and AMNH 5656, and further still in CMN 8803 and TMP 1999.55.292 (similar to the deeper embayments exhibited by *Agujaceratops* and *Utahceratops*).

The median bar is narrow and strap-like in "*Chasmosaurus russelli"* referred specimens CMN 2280, TMP 1983.25.1, and AMNH 5656, with the point of maximum constriction occurring in the posterior third, close to the point of contact with the posterior bar. There are no well developed lateral flanges that invade the parietal fenestrae (as seen in *Terminocavus*, Taxon C, or *Anchiceratops*). Godfrey and Holmes (1995) note that in "*C. russelli"* referred specimens CMN 2280, TMP 1981.19.175, and TMP 1983.25.1, the lateral bars do not fully enclose the fenestrae, with the squamosals therefore composing part of the mediolateral borders of the fenestrae. Godfrey and Holmes (1995) note that in AMNH 5656 the lateral bars do enclose the fenestrae.

Each referred specimen of *Chasmosaurus russelli* exhibits up to three pairs of epiparietals. Locus ep1 is positioned at the edge of the median embayment of the posterior bar in TMP 1983.25.1, AMNH 5656, and CMN 8803, but is slightly within the embayed area in CMN 2280 and TMP 1999.55.292 (i.e. more similar to *Utahceratops* and *Pentaceratops*, where ep1 is fully within the embayment). Locus ep1 is slightly anteriorly recurved in CMN 2280. The basal length of ep1 is slightly more elongate than ep2 and ep3 in some referred specimens of "*C. russelli"* (CMN 2280; TMP 1983.25.1; AMNH 5656), although it is not as extremely elongated as seen in specimens of *C. belli* (see below). In some specimens, locus ep1 exhibits a possible accessory process (as noted by Longrich, 2010), with a small bump present medial to a fused ep1 in TMP 1983.25.1, and a double process at the ep1 locus in CMN 8803 (to which no epiparietal is yet fused). The epiparietal at locus ep1 is a scalene triangle with the posterior apex of the triangle displaced laterally. Ep2 is of similar or smaller size than ep1 and is similarly shaped like a scalene triangle with the apex displaced laterally and pointing posterolaterally. Ep3 is slightly smaller than ep1 and 2 and is shaped like an isosceles triangle with the apex placed centrally. The apex of ep3 points laterally in TMP 1983.25.1 and AMNH 5656, but posterolaterally in CMN 2280 (especially on the left side).

Some characteristics indicate that "*Chasmosaurus russelli"* referred specimens AMNH 5656 and TMP 1983.25.1 represent immature individuals (more pointed epiparietals; unfused or pointed episquamosals, weak elongation of the squamosal, small size in the case of AMNH 5656). This may account for the slight differences between their parietal morphology and that of the most complete referred specimen, CMN 2280. Curiously, the fact that the smallest referred specimen of "*C. russelli"*, AMNH 5656, has parietal lateral bars that completely enclose the parietal fenestrae (whereas in all other specimens the lateral bars are open), suggests that the lateral bars may be closed early in ontogeny, but open up with maturity. This would be consistent with the hypothesis that parietal fenestrae open and enlarge through ontogeny (Brown et al., 2009; Scannella and Horner, 2010; Fastovsky et al., 2011; see main text for further discussion).

*Chasmosaurus belli* (including *Chasmosaurus* sp.)

Variation in parietal morphology is more notable among specimens referred to *Chasmosaurus belli* than in *C. russelli*. This is especially important concerning YPM 2016 (referred to *C*. sp. by Campbell et al., 2019) which in many ways is a morphological intermediate*.*

The parietal of *Chasmosaurus belli* is unlike referred specimens of *C. russelli* in that the lateral rami of the posterior bar are straight giving a more triangular or chevron shape to the parietal, rather than forming a rounded M-shape. In most specimens referred to *C. belli* the anteroposterior thickness of the posterior bar is equal from medial to lateral, with the exception of NHMUK R4948, which tapers slightly from medial to lateral. The posterior bar is relatively narrow anteroposteriorly, although less so than in *C. russelli*. The lateral rami of the posterior bar meet medially at an angle forming a broad shallow embayment, which mostly does not extend anteriorly beyond the posteriormost extent of the fenestrae (with the possible exception of slight overlap observed in AMNH 5402, although this specimen is distorted). The angle between the lateral rami is shallower than in *C. russelli*, and is relatively consistent at 149°–152° in *C. belli* holotype CMN 491, and referred specimens NHMUK R4948, ROM 843, and CMN 2245 (Fig. S11). However, YPM 2016 exhibits a posterior bar that is closer to straight, with the angle between the lateral rami at 167° (Fig. S11). This is interesting as it is therefore intermediate between typical *C. belli* specimens (149°–152°) and *Vagaceratops* (*Chasmosaurus*) *irvinensis*, which succeeds *C. belli* stratigraphically and has an almost horizontal posterior bar (177°; Fig. S11).

The median bar of *C. belli* is narrow and strap-like with the point of maximum constriction occurring in the posterior third, close to the point of contact with the posterior bar. There are no well developed lateral flanges that invade the parietal fenestrae (as seen in *Terminocavus*, Taxon C, or *Anchiceratops*).

The positioning and size of epiparietals varies more among referred specimens of *Chasmosaurus belli* than "*C. russelli"* (see Fig. S18). Understanding epiparietal morphology is critical as their correct identification provides additional synapomorphies linking *C. belli* specimens with more derived, stratigraphically younger taxa (*Vagaceratops* and *Kosmoceratops*), and helps highlight stratigraphic morphological trends over 2–4 million years. It is likely that specimens of *C. belli* exhibit three epiparietal loci (ep1–ep3) on each lateral ramus of the posterior bar, however, assignment of numbers to epiparietal loci is confounded by variation among specimens (especially regarding ep2 and ep3), and most specimens are either incomplete or lack epiparietal fusion (possibly due to being non-adult ontogenetic status; Horner and Goodwin, 2008). In all specimens referred to *C. belli*, locus ep1 is occupied by an elongate ridge, recurving anteriorly, occupying 50% or more of each lateral ramus of the posterior bar. Typically the ep1 ridge is relatively unornamented, and does not bear discernible epiparietals (e.g. CMN 491; NHMUK R4948, ROM 843, CMN 2245, AMNH 5402). This may be an ontogenetic artifact, but given the probable most mature status of ROM 843 (see Campbell et al., 2016), it is possible that prominent discrete epiparietals might not be present at all. As such, this region might therefore not even be considered an epiparietal locus. However, in YPM 2016 the ridge bears the rugose texture characteristic of epiossifications, forming an undulating surface comprised of 3 to 4 processes (Lehman, 1998) or 4 processes (Campbell et al., 2019). YPM 2016 is therefore intermediate in morphology between other specimens of *C. belli* (e.g. ROM 843) and that of *Vagaceratops* (*Chasmosaurus*) *irvinensis* (and *Kosmoceratops richardsoni*) in which the processes are more developed and differentiated into individual spikes (Campbell et al., 2019; see later).

The size and position of loci ep2 and ep3 is particularly variable. In AMNH 5402,YPM 2016, and probably CMN 2245, the largest epiparietal is located immediately medial to the parietal-squamosal contact, occupying locus ep3 and oriented posterolaterally, with ep2 more weakly developed (if present at all). However, in ROM 843, the epiparietal immediately medial to the squamosal contact is small and described as "unfused (and presumably recently attached)" by Godfrey and Holmes (1995; p. 738). It is not clear if this is ep3 (e.g. as shown by Campbell et al., 2016), or (as suggested by Godfrey and Holmes, 1995) an additional fourth epiparietal (ep4) peculiar to ROM 843. Godfrey and Holmes (1995) suggest that the particularly large triangular epiparietals positioned between ep1 and the possible ep4 might represent ep2 and ep3 fused together. This hypothesis is supported by the double-peaked epiparietal exhibited on the left side of ROM 843 (but not the right side; Godfrey and Holmes, 1995). An alternative interpretation could be that this large epiparietal is locus ep2, and this would be consistent with its posterior orientation (comparable with "*C. russelli*" specimen CMN 2280). If this is indeed the case, then the small epiparietal immediately adjacent to the parietal-squamosal contact may truly represent ep3. This intriguing suggestion may mean that ep3 is actually lost in the presumably more derived CMN 2245, YPM 2016, and AMH 5402 (and later, *Vagaceratops* and *Kosmoceratops*), which otherwise exhibit an elongate ep1 locus and a large corner epiparietal (?ep2). However, although ROM 843 is often viewed as a typical example of *C. belli* (e.g. Godfrey and Holmes, 1995) it is unusual in the number and morphology of epiparietals for loci other than ep1. It is possible that this is an ontogenetic artifact, as ROM 843 is noted as being particularly large and might therefore represent an aged individual.

It is also worth noting that both AMNH 5402 and YPM 2016 were both moved out of *C. belli* by Campbell et al. (2019). Given the similarity between these two specimens and CMN 2245, it is not clear why CMN 2245 was left in *C. belli*.

### *Vagaceratops* (*Chasmosaurus*) *irvinensis* and *Kosmoceratops richardsoni*

Holmes et al. (2001) described *Chasmosaurus irvinensis* from two relatively complete skulls (holotype CMN 41357; referred specimen TMP 1987.45.1) and an exploded skull (TMP 1998.102.8; of which only a fragment of a parietal was diagnosable) from the Dinosaur Park Formation, Alberta. *Chasmosaurus* *irvinensis* was recovered as the sister taxon of *C. belli* by Holmes et al. (2001). Later, Sampson et al. (2010) described the new taxon *Kosmoceratops richardsoni* (now known from two specimens; holotype UMNH VP 17000; and a smaller referred specimen, UMNH VP 16878; Loewen et al., 2013) from the Kaiparowits Formation, Utah*,* and renamed *C*. *irvinensis* as *Vagaceratops* *irvinensis* to avoid paraphyly issues; their phylogenetic analysis placed [*Vagaceratops* + *Kosmoceratops*] as a separate clade that was recovered as much more derived than the clade comprising other species of *Chasmosaurus* (*C. belli* and *C. russelli*). Longrich (2014; using a different character matrix) recovered *V. irvinensis* as the sister taxon to *C. belli* (ie. the same relationship as recovered in the original analysis by Holmes et al., 2001), and therefore returned the taxon name to *C. irvinensis* (as did Campbell et al., 2019)*.* Notably, Longrich's analysis does not recover *Kosmoceratops* as the sister taxon to *Vagaceratops*. Although here we agree with Holmes et al. (2001) and Longrich (2014) that *V. irvinensis* is probably a derived form of *Chasmosaurus* (based on strong similarity of the parietal), for the same reason it is considered likely that *Kosmoceratops* is the sister taxon of *V. irvinensis* (as recovered by Sampson et al., 2010, and in phylogenetic analyses in this paper based on the Sampson et al., 2010 character matrix). For the time being, it is probably desirable to maintain the name *Vagaceratops* as it seems likely that the results of phylogenetic analyses will remain unstable for the foreseeable future, pending more detailed critical analysis of character matrices, and the description of new specimens of basal chasmosaurines.

In 2014, Longrich reassigned CMN 8801 (a large, nearly complete face lacking the frill and brow horns, although it is not clear if the brow horns have been broken or resorbed as is typical for many basal chasmosaurines) to *Kosmoceratops* n. sp. The specimen was previously designated as the paratype of *C. russelli* by Sternberg (1940), but considered only as Chasmosaurinae sp. by Godfrey and Holmes (1995) due to the lack of the posterior part of the frill. Better understanding of morphological characters in the anterior half of the skull (especially the premaxilla, and change through ontogeny) may make a more reliable referral possible in the future. Finally, it is worth noting here that Longrich (2014) states that the stratigraphic position of the CMN 8801 locality (quarry no. 63) had not been measured, and suggested that it occurred ~24 m above the Oldman - Dinosaur Park Formation contact. However, the stratigraphic position of CMN 8801 was stated by Godfrey and Holmes (1995) as 23.04 m above the Oldman - Dinosaur Park Formation contact (although Godfrey and Holmes do not state whether or not these are direct measurements, or extrapolations based on topographic height).

**Stratigraphy**

*Vagaceratops* was collected from the uppermost part of the Dinosaur Park Formation, Alberta, just below the Lethbridge Coal Zone (Holmes et al., 2001; Campbell et al., 2019). The holotype (CMN 41357) and one of the referred specimens (TMP 98.102.8) were found outside of Dinosaur Provincial Park, so their stratigraphic position is not as precisely known as the third specimen (TMP 1987.45.1). Holmes et al. (2001; p. 1433) state that the holotype (CMN 41357) derives from a layer "within 20m of the Lethbridge Coal Zone". The referred specimens derive from "within 20m and 10m, respectively, of the Lethbridge Coal Zone" (Holmes et al., 2001; p. 1433) although it is not made clear which of the referred specimens is which. However, Currie et al. (2005), Mallon et al. (2014), and Campbell et al., (2019) provide some stratigraphic data for the referred skull TMP 1987.45.1, showing the quarry (no. 184) at 712 m above sealevel, 50.4 m (± 7 m) above the Oldman - Dinosaur Park formational contact, or ~54m above the Oldman - Dinosaur Park formational contact (respectively). This places *V. irvinensis* ~11.1 m below a 76.10 Ma bentonite (which occurs 61.5 m above the formational contact; 3.5 m into the Lethbridge Coal Zone) and 24.4 m above a 76.39 Ma bentonite (occurring at 36 m above the formational contact; dates from Eberth, 2011; see Fowler, 2017).

The stratigraphic positions of *Kosmoceratops* specimens were plotted by Loewen et al. (2013) relative to a series of radiometrically dated bentonites, the precise positions and ages of which are most recently summarized in Roberts et al. (2013). The holotype (UMNH VP 17000) was recovered ~170 m above the base of the Kaiparowits Formation between a 76.46 Ma bentonite at 80 m, and a 75.97 Ma bentonite at 190 m. Referred specimen UMNH VP 16878 was collected at ~200 m above the base of the Kaiparowits Formation, between the 75.97 Ma bentonite at 190 m, and a 75.51 Ma bentonite at 420 m. Thus both specimens occur close to the 75.97 Ma datum.

*Vagaceratops* is therefore shown to occur at ~76. 2 Ma (no younger than 76.1 Ma), and *Kosmoceratops* at ~76–75.9 Ma. Thus the taxa do not overlap stratigraphically.

**Morphology**

The lateral rami of the posterior bar are virtually horizontal in *Vagaceratops*, meeting at an angle of 177° in the holotype CMN 41357 (Fig. S11), and therefore lacking any kind of embayment. In the *Kosmoceratops* holotype (UMNH VP 17000)*,* this angle is more acute, being measured as 123° based on the drawing of the skull in dorsal view in Sampson et al. (2010). However, this is probably slightly exaggerated as an artifact of the curvature of the parietal, as when the skull is observed in anterior view the posterior bar appears more straight (indeed, "transverse parietal bar straight" is a character listed by Sampson et al., 2010, in the diagnosis). In a smaller (presumably immature) specimen (UMNH VP 16878) referred to *Kosmoceratops* by Loewen et al. (2013), the lateral rami meet at 142°. Thus, the posterior bar of *Kosmoceratops* is slightly embayed, to a similar extent to most specimens of *Chasmosaurus belli* (149–167°).

The median bar is only preserved in the holotypes of *Vagaceratops* (CMN 41357) and *Kosmoceratops* (UMNH VP17000). In *Vagaceratops* the median bar is broader than in specimens of *Chasmosaurus russelli* or *C. belli*, but less broad than *Kosmoceratops*. The point of maximum constriction of the median bar is difficult to assess in *Vagaceratops* as the median bar is partly broken on both sides. The reconstruction of *Kosmoceratops* in Sampson et al. (2010) shows the point of maximum constriction occurring in the posterior third. *Vagaceratops* exhibits lateral flanges of the median bar which are similarly intermediate between the condition in *C. belli* and the more developed flanges of *Kosmoceratops*. In *Vagaceratops* the lateral bars fully enclose the parietal fenestra on the left side of both the holotype and referred specimen TMP 1987.45.1, but on the right side of the holotype it is discontinuous, with ~ the squamosal forming ~5 cm of the lateral border of the fenestra (Holmes et al., 2001). In *Kosmoceratops* (UMNH VP 17000) the lateral bar fully encloses the fenestra on the right side, but the left side is not preserved. In *Vagaceratops* the lateral bars are thin, as in *C. belli* and *C.* sp., whereas in *Kosmoceratops* they are much broader, more similar to *Anchiceratops*, and more derived chasmosaurines.

In the figures provided by Holmes et al. (2001), the parietal fenestrae (and squamosals) of *Vagaceratops* appear anteroposteriorly short compared to *Chasmosaurus russelli* and *C. belli* (see Fig. S18). This is an artifact of the higher angle at which the frill is held relative to the face, which was specifically noted by Holmes et al., 2001, and also observed in *Kosmoceratops* (Sampson et al., 2010). Holmes et al. (2001) figure the *Vagaceratops* frill in both anterior and dorsal views, but neither of these is comparable to the more typical "plan-type" view where the anteroposterior length of the squamosal lies flat on the resting surface. Consultation of photographs of the *Vagaceratops* holotype (CMN 41357) demonstrate that when oriented flat, the fenestrae are much longer and comparable to those of *C. belli* specimen YPM 2016 (see later).

### Reconfiguration of epiparietal numbering system

The configuration of epiparietals and posterior episquamosals for *Vagaceratops* and *Kosmoceratops* differs among the original descriptions (Holmes et al., 2001; Sampson et al., 2010; also Loewen et al, 2013; Fig. S18). The identification of these epiossifications is important in phylogenetic analysis, and here we have revised the numbering systems offered by previous authors based on comparisons with specimens of *Chasmosaurus belli* and *C*. sp.

In the description of *Vagaceratops* (*Chasmosaurus*) *irvinensis*, Holmes et al. (2001) describe two specimens which preserve the squamosal-parietal contact (holotype CMN 41357 and referred specimen TMP 87.45.1). In the holotype, the parietal is described as exhibiting five epiparietals on each side (numbered ep1, medialmost through ep5, lateralmost; Fig. S18; Holmes et al., 2001). Ep1–4 are described as being imperceptibly fused to one another, and formed of two thin laminae curving anteriorly from the parietal posterior bar (Holmes et al., 2001). In contrast, referred specimen TMP 87.45.1 only preserves an ep4 (ep1–3 are missing) but it is described as well-preserved and triangular (Holmes et al., 2001). In the holotype CMN 41357, the lateralmost epiparietal (ep5) is "stout, shield-shaped, and forms a firm broad attachment to the [lateral ramus of the parietal posterior] bar adjacent to the parietal-squamosal suture" (Holmes et al., 2001; p. 1429). Ep5 is missing in TMP 87.45.1, with an oval facet in its place to which presumably the unfused epiparietal would have attached in life. It is important to note that ep5 is mounted on the posterolateralmost corner of the parietal which forms a small triangular extension over the posterior end of the squamosal (especially clear in Fig. S18H). In the holotype CMN 41357, the ep5 locus very closely abuts the posteriormost end of the squamosal, such that it might be considered as an eps (epi-parietal-squamosal; referring to an epiossification that straddles the parietal-squamosal contact). This is significant as the large hook-shaped posterolateralmost frill epiossification present in *Kosmoceratops* is in a similar position, but is considered as the posteriormost episquamosal (es1; Fig. S18C) by Sampson et al., (2010).

The configuration of epiparietals in *Kosmoceratops richardsoni* (Sampson et al., 2010) is strikingly similar to *Vagaceratops*, although the numbering system is slightly different (Fig. S18). As in *Vagaceratops*, four processes project anteriorly from the posterior border of the parietal posterior bar. However, in *Kosmoceratops* these processes form elongate spikes, whereas in *Vagaceratops* they are more modest triangular processes. It is notable that the spikes are relatively even more elongate in juvenile *Kosmoceratops* UMNH VP 16878 than in the larger more mature holotype UMNH VP 17000. These are numbered as ep1 through ep3, with what is considered as ep4 in *Vagaceratops* labeled as an eps. The epiossification present at the posterolateralmost corner of the *Kosmoceratops* frill is labeled as es1, (ie. the first episquamosal), rather than ep5 (as in *Vagaceratops*; Holmes et al., 2001). In their character matrix, Sampson et al. (2010) renumber the frill epiossifications of *Vagaceratops* to match *Kosmoceratops*. Here we agree that it is probable that the epiossifications are homologous, but disagree with the numbering system.

Based on comparison to *Chasmosaurus*, especially *C.* sp specimen YPM 2016, here it is proposed that previous epiossification numbering systems of *C*. sp *Vagaceratops* and *Kosmoceratops* should be revised (Fig. S18). This is important as epiossification numbering directly affects 17 phylogenetic characters, and the proposed renumbering suggests previously unrecognized synapomorphies shared by *C. belli*, *C*. sp. and *Vagaceratops*.

Campanian chasmosaurines typically exhibit three paired epiparietals, which are numbered from the midline laterally as ep1 to ep3 on each side, making a total of six. This is easily observed in *C. russelli* specimen CMN 2280 (Fig. S18D) where three epiparietals with elongate bases are attached to the posterior margin of the parietal posterior bar.

The positioning and numbering of epiparietals in *C. belli* (and *C*. sp; Campbell et al, 2019) is not as obvious, and varies slightly among individuals (as described above; Fig. S18 E, F).

An elongate anteriorly recurving ridge occupies 50% or more of each lateral ramus of the posterior bar. This is labeled as "p1" by Campbell et al. (2016) on ROM 843 and CMN 491 (the holotype of *C. belli*), and is clearly observed in CMN 2245 and NHMUK R4948 (see figure 10 in Maidment and Barrett, 2011). In most specimens this ridge is relatively unornamented; in some specimens this is probably because epiparietals had not yet fused on to this part of the parietal (e.g. the probable immature specimen CMN 2245, see above). However, in YPM 2016 the ridge bears the rugose texture characteristic of epiossifications, forming an undulating surface comprised of three to four short processes (abutted against a large triangular epiparietal present immediately adjacent to the squamosal suture). Campbell et al. (2016; 2019) label these processes individually as ep1–4 with the large triangular process immediately adjacent to the squamosal labeled as ep5. Campbell et al., 2019 further suggested that small bumps on the posterior bar of AMNH 5402 are loci for additional processes and similarly label these ep 1–4 loci, using this as justification for reassigning YPM 2016 and AMNH 5402 from *C. belli* to *C*. sp. (but do not consider the similar specimen CMN 2245). Here we agree with the proposal (Campbell et al., 2019) that the multiple processes present on YPM 2016 (and possibly AMNH 5402) are homologous with the first three (or possibly four) processes of *Vagaceratops,* extend this to *Kosmoceratops*, and therefore require revision of the numbering system.

The question remains as to what to call these processes, as the method chosen infers homology with the same numbered process in other taxa. Campbell et al. (2016; 2019) simply number the processes out from medial to lateral, the standard method for ceratopsids and previously employed for *Vagaceratops* and *Kosmoceratops* (Holmes et al., 2001, Sampson et al., 2010). However, as characters code homologies, then this method therefore proposes that the lateralmost epiparietal (ep3) in most specimens of *C. belli* and *C. russelli* (indeed, most chasmosaurines) is in fact homologous with an epiparietal that occurs in the middle of each lateral ramus (ep3) in *C*. sp. *Vagaceratops* and *Kosmoceratops*, rather than the lateralmost epiparietals (ep5) in those specimens.

Under our proposed renumbering system, the lateralmost epiparietal is always ep3, as befits its morphology and position. We suggest the origin of the medialmost three or four epiparietals (sensu Campbell et al., 2019) is expansion and subdivision of the ep1 locus. This is consistent with the observation that from "*C. russelli*" to "*C. belli*" the ep1 locus expanded laterally to occupy the majority of the posterior bar (see ROM 843 and CMN 491 in Campbell et al., 2016).

Locus ep3 is reidentified as the lateralmost epiparietal in both *Vagaceratops* (previously identified as ep5 by Holmes et al., 2001; Campbell et al., 2016; 2019) and *Kosmoceratops* (previously identified as es1 by Sampson et al., 2010, and Loewen et al., 2013). The epiparietal in this position abuts the squamosal and is in the expected position for ep3. Locus ep2 is designated to the epiparietal that occurs between the elongate ep1 and ep3 (see Fig. S18). This is the only epiparietal to be preserved in *Vagaceratops* referred specimen TMP 87.45.1, supporting its designation as a discrete epiossification.

It is acknowledged that this revision might be controversial, but it is the best representation of the hypothesis that the epiparietals of *Chasmosaurus* sp., *Vagaceratops* and *Kosmoceratops* are homologous to the three paired epiparietals of *Chasmosaurus russelli.* Perhaps there is a better system (maybe abandoning a numbering system completely), but this is beyond the scope of the current work.

## Agujaceratops (Chasmosaurus) mariscalensis

Lehman (1989) described *Chasmosaurus mariscalensis* from a range of material collected from the Aguja Formation, Texas. Most of the cranial material (including that of the holotype, UTEP P.37.7.086; an associated braincase, brow horn, maxilla, and dentary) was recovered in the late 1930's from locality WPA1 (Works Progress Administration), a multi-individual bonebed which occurs low in the Upper Shale Member (Lehman, 1989; Lehman and Busbey, 2007). However, the only specimen figured by Lehman (1989) that preserves the diagnostic midline of the parietal posterior bar is UTEP P.37.7.065 (an isolated fragmentary parietal associated with two humeri) which was collected from locality WPA3 (shown as stratigraphically equivalent to WPA1 by Lehman, 1989; Lehman and Busbey, 2007). Although Lehman (1989) mentions fragmentary parietal specimens from the WPA1 bonebed (UTEP P.37.7.063, 064, 089, and 093), these are neither described nor figured. In 1991, a nearly complete chasmosaurine skull was collected from higher in the Upper Shale Member of the Aguja Formation (Lehman and Busbey, 2007). This skull (TMM 43098-1) was referred to *C. mariscalensis* by Forster et al. (1993). The taxon was renamed as *Agujaceratops* *mariscalensis* by Lucas et al. (2006), which has been followed by most workers since, and is the name used in the current study. The near-complete skull TMM 43098-1 was designated as the type specimen of a second species, *A. mavericus* by Lehman et al. (2017), who also described a number of new parietal fragments (TMM 46501-1) from the same stratigraphic level as *A. mariscalensis*.

**Stratigraphy**

Lehman (1989) states that quarries WPA1 and WPA3 are located in the lower part of the Upper Shale Member of the Aguja Formation, "less than 70 m" above the underlying Terlingua Creek Sandstone, upon which the Upper Shale Member rests. A series of more detailed measured sections illustrated by Lehman and Busbey (2007; their fig. 35) show WPA1 as occurring immediately (<5m) above the Terlingua Creek Sandstone, and WPA3 as being stratigraphically equivalent (although the Terlingua Creek Sandstone is not present at the WPA3 section). Alternatively, Lehman et al. (2016) show WPA1 and WPA3 as ~5m and ~20m above the top of the Terlingua Creek Sandstone (respectively), noting that these sites are subject to some uncertainty due to igneous intrusions and variable unit thickness in these areas. The additional parietal fragments described by Lehman (2016) were collected from ~15m above the top of the Terlingua Creek Sandstone. By contrast, the locality from which the more complete skull (TMM 43098-1) was recovered is from much higher in the Upper Shale Member, positioned among a series of channel sandstones ~50–70 m (Forster et al., 1993; Lehman and Busbey, 2007; their fig. 19) or perhaps as high as 110m (Lehman et al., 2016) above the top of the Terlingua Creek Sandstone.

The Terlingua Creek Sandstone is dated by the presence of the ammonite *Baculites mclearni* (Rowe et al., 1992; 80.67–80.21 Ma; lowermost part of the middle Campanian; Ogg & Hinnov, 2012). As noted previously (see *Bravoceratops* entry), two U-Pb radiometric dates related to phreatomagmatic volcanism have been published as from within the Upper Shale Member of the Aguja Formation; 76.9 +/- 1.2 Ma (Befus et al., 2008), and 72.6 +/- 1.5 Ma (Breyer et al., 2007), although it is likely that the volcanism occurred after the Upper Shale Member had been deposited (Befus et al., 2008; Fowler, 2017).

The age of the quarries from which *A. mariscalensis* material was collected is not precisely known, but can be constrained as to between 80.21 and 76.9 Ma (probably closer to the older date; see above). It is therefore possible that *A. mariscalensis* is among the stratigraphically oldest known chasmosaurines. The higher stratigraphic position of TMM 43098-1 demonstrates that this specimen is younger than the WPA1 and WPA3 specimens, although by how much is difficult to assert, especially given that it occurs within a stratigraphic interval illustrated as being sand dominated, perhaps suggestive of having been deposited under a low accommodation setting rather than the high accommodation setting of the mud-dominated parts (e.g. Atchley et al., 2007). Here we are therefore not confident in asserting a stratigraphic placement for TMM 43098-1.

**Morphology**

As the only figured specimen which includes the diagnostic midline of the parietal posterior bar, only UTEP P.37.7.065 can be compared to *Navajoceratops* and *Terminocavus*. UTEP P.37.7.065 is fragmentary and in three pieces (see Fig. S10). The largest piece comprises the middle part of the posterior bar, the medialmost parts of the lateral rami, and the posterior end of the median bar. Two other pieces comprise the lateralmost edge of the left lateral ramus of the posterior bar, and part one of the lateral bars. The lateral rami of the posterior bar are narrow struts of bone, meeting at an angle of 80° (Fig. S10) forming a median embayment that extends anterior to the posteriormost edge of the parietal fenestrae. The lateral rami are broken, but the lateral fragment is of similar anteroposterior thickness to the medial parts.

The median bar narrows anteriorly; the point of maximum constriction is unknown, but it is not immediately below the confluence of the lateral rami of the posterior bar; i.e., it is not similar to species of *Chasmosaurus*, being more like the condition in specimens of c.f. *Pentaceratops sternbergii*. The median bar bears small lateral flanges that extend into the parietal fenestrae. Although Lehman (1989) suggests that these are present on the ventral side of the median bar, it would be more consistent with other chasmosaurine specimens if this was actually the dorsal side. The fragment of the lateral bar is too incomplete to establish to what extent it may have enclosed the parietal fenestrae.

A single epiparietal locus is preserved at the edge of the median embayment on the right side, this is presumed to represent ep1, although no epiparietal is fused to the locus; another epiparietal locus is preserved on the lateral fragment, and is presumed to represent ep3. Orientation of the loci is not discernible from published images (Lehman, 1989).

The three or four parietal fragments (TMM 46501-1) described by Lehman et al. (2016) do not fit together in an obvious way and are variably hypothesized to represent ep1–4.

**Comparisons**

UTEP P.37.7.065, the holotype of *Agujaceratops*, exhibits a mosaic of characters variably seen in other relatively basal chasmosaurines. The median embayment is deeper than in *Chasmosaurus*, *Vagaceratops*, and *Kosmoceratops,* but similar to *Utahceratops* and cf. *Pentaceratops sternbergii*. Although the point of maximum constriction of the medial bar is not precisely knowable (due to incompleteness), it does not occur immediately anterior to the contact with the posterior bar, as in most *Chasmosaurus* specimens (especially, *C. belli*). In contrast, the position of ep1 outside of the embayed area is more consistent with *Chasmosaurus* than *Utahceratops*, *Pentaceratops*, and more derived forms. Indeed, UTEP P.37.7.065 bears strongest similarity to CMN 8803 (Fig. S11), an aberrant isolated midline parietal fragment first referred to *C. russelli* by Sternberg (1940), then to *Mojoceratops perifania* by Longrich (2010). Given the current revision of *C. russelli*, the taxonomic affinity of CMN 8803 is unclear, so the significance of its similarity to UTEP P.37.7.065 is difficult to assess. It is tempting to suggest that UTEP P.37.7.065 may represent a more basal form of the *Utahceratops* clade, which would be consistent with its biogeography and probable stratigraphic occurrence. However, testing of such a hypothesis requires the discovery of better material.

**Discussion**

It is not the intent of this current study to revise the diagnoses of every chasmosaurine. Nevertheless, there are important taxonomic issues affecting *Agujaceratops mariscalensis* that need to be highlighted, especially because of its inclusion in phylogenetic analyses.

Firstly, no overlapping diagnostic elements were described between the holotype bonebed material (site WPA1), and the referred parietal midline specimen UTEP P.37.7.065 (site WPA3). Lehman (1989) does note that some parietal fragments were collected from WPA1 (UTEP P.37.7.063, 064, 089, and 093), but these are not described or figured, and no justification is given for referring UTEP P.37.7.065 to *A. mariscalensis*. Two humeri were collected from WPA3 and are comparable to humeri from WPA1 in that they are more slender than other ceratopsids with a deltopectoral crest that is more restricted than other chasmosaurines (Lehman, 1989). However, the taxonomic value of chasmosaurine postcrania is not well understood, and it would be tentative at best to assign the WPA3 material to *A. mariscalensis* on the basis of postcrania alone. Therefore, it is not technically possible for UTEP P.37.7.065 to be referred to the same taxon as the holotype, especially as it is not from the same multi-individual bonebed as the other specimens. However, given that both localities occur at the same stratigraphic level, low in the Aguja Formation, then it is at least possible if not likely that the specimens belong to the same taxon. It is also notable that the material from the WPA3 quarry (parietal, two humeri) is from a larger individual than all or most material from the holotype bonebed WPA1, suggesting that it may be from a more mature individual.

A second problem is that the characters used by Lehman (1989) to diagnose *Agujaceratops mariscalensis* include some that are now recognized as being either undiagnostic or indicative of immature status rather than taxonomic distinction. All specimens from the WPA1 bonebed (including the largest purportedly adult specimens; Lehman, 1989) exhibit morphological characteristics indicative of varying degrees of immaturity in chasmosaurines (small size; unelongated premaxilla; posteriorly curving postorbital horncores; short postorbital horncores relative to long-horned chasmosaurines; relatively short and broad squamosals; fewer episquamosals than larger individuals with elongate squamosals; pointed episquamosals; weakly fused or unfused frill epiossifications; crenulated squamosal lateral margin; Lehman, 1990; Godfrey and Holmes, 1995; Horner and Goodwin 2006; 2008; Scannella and Horner, 2010). Indeed, immaturity of the WPA1 material was recognized by Forster et al. (1993) in their description of the more complete skull (TMM 43098-1; see below). The issue with the immaturity of the WPA1 material is that juvenile dinosaurs typically exhibit different character states than adults of their own species, often appearing more basal (Rozhdestvensky, 1965; Fowler et al., 2011; Campione et al., 2013). Thus, the diagnosis of *Agujaceratops mariscalensis* by Lehman (1989) includes characters that are consistent with the immature status of the type material rather than diagnostic of an adult (short broad squamosal; fewer episquamosals than larger individuals with elongate squamosals; premaxilla without posterodorsal extension, i.e. premaxilla is anteroposteriorly short). Other characters used by Lehman (1989) to diagnose *A. mariscalensis* (supraorbital horncores longer than in adults of other species of *Chasmosaurus*; maxilla without lateral horizontal shelf) may distinguish *A. mariscalensis* from species of *Chasmosaurus* (as was Lehman's intent), but do not distinguish *A. mariscalensis* from other long-horned chasmosaurines such as *Pentaceratops sternbergii* (indeed, Lehman, 1989, notes the similarity of *A. mariscalensis* to *P. sternbergii*, suggesting that they may be closely related). Therefore, the diagnosis of *A. mariscalensis* by Lehman (1989) is invalid. An attempt to rectify this is provided by Forster et al. (1993) in their description of a more complete skull (TMM 430981-1), however this specimen has its own problems (see next paragraph).

The third problem is that there is little justification for referring TMM 43098-1 (the partial skull described by Forster et al., 1993) to the same taxon (i.e. *A. mariscalensis*) as material from the WPA1 (holotype) or WPA3 (referred parietal) quarries. Firstly, as TMM 43098-1 does not preserve any part of the parietal, it cannot be compared with the WPA3 parietal (UTEP 037.7.065). On the basis of the new skull, Forster et al. (1993) rediagnose *A. mariscalensis*. However, characters in the new diagnosis and in the discussion of Forster et al. (1993) are either not observable in the WPA1 type material, represent symplesiomorphies, or are known to vary ontogenetically.

Character 1, a "strong anteromedian nasal process extending between the premaxillae on the internarial bar" (Forster et al., 1993; p.162) is an unusual feature so far reported only in TMM 43098-1 (among chasmosaurines). However, this condition is not recorded for the type WPA1 bonebed material as no nasals were recovered. Furthermore, the single known premaxilla present in the WPA1 material is not figured in medial view by Lehman (1989), so any recessed articulation surface that might have received an anteromedian process of the nasal has not been observed, and no comment on this is provided by Forster et al. (1993). The holotype of *Pentaceratops sternbergii* (AMNH 6325) has similar grooves visible on the dorsal surface of the rostrum, suggesting that it too might exhibit a tongue-like extension of the nasals extending a short distance anteriorly between the premaxillae. However, this is difficult to see in the AMNH mount as it is covered by reflective glass. Regardless, this character is not demonstrable in the type material and so cannot be used to refer TMM 43098-1 to the same taxon as the WPA1 material.

Character 2, "erect supraorbital horncores that attain an angle of 85° to the maxillary tooth row in adults" (Forster et al., 1993; p.162) is observed in TMM 43098-1, and some of the larger postorbital horns from the holotype WPA1 quarry (UTEP P.37.7.082; 086, and 091; Lehman, 1989). However, the apparent erectness is an artifact of the posterior curvature of the horn tip (an indication of immaturity; Horner and Goodwin, 2006); when measured at the base (Fig. S19), the postorbital horns are equally erect in the holotype of *Pentaceratops sternbergii* (AMNH 6325). Since equally erect postorbital horns are exhibited by other Campanian chasmosaurines then this character cannot be considered autapomorphic of *Agujaceratops mariscalensis*, and cannot be used to refer the TMM 43098-1 skull to the same taxon as the WPA1 material.

Character 3, "squamosal with a convex lateral frill margin" (Forster et al., 1993; p.162) is an important morphological feature of TMM 43098-1, but whether or not it is diagnostic (and can therefore be used to unite TMM 43098-1 with WPA1 material) is clouded by variability in the WPA1 material, and the fact that this character is known to vary ontogenetically in other chasmosaurines. In two of the WPA1 squamosal fragments, the posterior end has a lateral margin that is relatively straight (UTEP P37.7.046; 052; Lehman, 1989), which might indicate that their morphology is unlike that of TMM 43098-1; however, other specimens from the WPA1 bonebed do have a lateral margin that is convex (e.g. UTEP P37.7.062; Lehman, 1989). Convexity of the lateral margin of the squamosal is influenced by ontogeny in other chasmosaurines; in the derived chasmosaurine *Triceratops*, the squamosal is short and broad for most of ontogeny, and similarly has a convex lateral margin (Scannella and Horner, 2010). If the WPA1 material is all immature then it is not possible to determine whether the lateral convexity observed in some specimens is due to ontogeny or a genuine taxonomic feature. Similarly, the condition in TMM 430981-1 may therefore be more reflective of ontogeny rather than phylogeny, supported by the posteriorly curving postorbital horns which are also indicative of immaturity (Horner and Goodwin, 2006). Nevertheless it is interesting that the squamosal of TMM 43098-1 is not as "bladed" (elongated) as squamosals of other Campanian chasmosaurines such as *Chasmosaurus belli* and *Pentaceratops sternbergii*. This complicates matters regarding *A. mariscalensis* as the other WPA1 squamosals are all from immature individuals which might be expected to have a short broad squamosal with a convex lateral margin.

As a result of the invalid diagnosis of *Agujaceratops mariscalensis*, TMM 43098-1 cannot be shown to share any diagnostic features with the type (WPA1) or referred (WPA2) material. TMM 43098-1 is also from a stratigraphic level that is ~50–70 m higher in the Aguja Formation than the WPA1 and WPA2 quarries, over which there is a change in depositional style from mud- to sand-dominated; this might reasonably be interpreted as representing a change in overall depositional conditions including available accommodation space, which further suggests that a considerable amount of time might be represented by the stratigraphic gap between the specimens. It has been shown (Horner et al., 1992; Mallon et al., 2012; Scannella et al., 2014; Fowler, 2017) that dinosaur species (especially ceratopsids) evolve rapidly over as little as a few hundred thousand years, so it might not be expected that chasmosaurine morphospecies would be the same at the bottom and top of the Upper Shale Member. Therefore it does not seem appropriate to assign TMM 43098-1 to *A. mariscalensis*.

This proposal creates a problem in that the phylogenetic dataset of Sampson et al. (2010; and presumably, subsequent modifications) use an amalgam of "TMM 43098-1 and UTEP bonebed specimens" (in their supporting information, p. 2). Use of an amalgam of immature (type material from WPA1) and mature material (WPA2 referred parietal and TMM 430981-1) which cannot be demonstrated to be from the same taxon, is clearly not ideal, and may explain the disruptive nature of *Agujaceratops* on cladistic analysis (see main text). The solution to this problem is probably to ignore the probable juvenile material from the WPA1 bonebed, and to code TMM 43098-1 and UTEP P.37.7.065 separately.

# REFERENCES

Atchley, S. C., L. C. Nordt, and S. I. Dworkin. 2004. Eustatic Control on Alluvial Sequence Stratigraphy: A Possible Example from the Cretaceous-Tertiary Transition of the Tornillo Basin, Big Bend National Park, West Texas, U.S.A. Journal of Sedimentary Research 74:391–404.

Bauer, C. M. 1916. Contributions to the Geology and Paleontology of San Juan County, New Mexico: Part 1. Stratigraphy of a Part of the Chaco River Valley. US Government Printing Office, pp.

Befus, K. S., R. E. Hanson, T. M. Lehman, and W. R. Griffin. 2008. Cretaceous basaltic phreatomagmatic volcanism in west Texas: maar complex at Pena Mountain, Big Bend National park. Journal of Volcanology and Geothermal Research 173:245–264.

Brown, B. 1914. *Anchiceratops*, a new genus of horned dinosaurs from the Edmonton Cretaceous of Alberta. Bulletin of the American Museum of Natural History 33:539–548.

Brown, B. 1933. A new longhorned Belly River ceratopsian. American Museum Novitates 669:1–3.

Brown, C. M. 2009. Pattern and transition of surficial bone texture of the centrosaurine frill and their ontogenetic and taxonomic implications. Journal of Vertebrate Paleontology 29:132–141.

Brownfield, M. E., and E. A. Johnson. 2008. The Yampa Bed-A Regionally Extensive Tonstein in the Williams Fork Formation, Northwestern Piceance Creek and Southern Sand Wash Basins, Colorado. Geological Survey (US), pp.

Campbell, J., M. J. Ryan, C. Schröder-Adams, R. B. Holmes, and D. C. Evans. 2013: A specimen-based phylogenetic analysis of the chasmosaurine ceratopsid Chasmosaurus (Ornithischia) from the Upper Cretaceous (Campanian) Dinosaur Park Formation of western Canada suggests the validity of only one species. Society of Vertebrate Paleontology 73rd Annual Meeting, Abstracts of Papers, Los Angeles, 2013.

Campbell, J.A., Ryan, M.J., Holmes, R.B., Schröder-Adams, C.J., 2016. A re-evaluation of the chasmosaurine ceratopsid genus *Chasmosaurus* (Dinosauria: Ornithischia) from the Upper Cretaceous (Campanian) Dinosaur Park Formation of western Canada. PLoS ONE 11, e0145805. <https://doi.org/10.1371/journal.pone.0145805>

Campbell, J.A., Ryan, M.J., Schröder-Adams, C.J., Evans, D.C., Holmes, R.B., 2018. New insights into chasmosaurine (Dinosauria: Ceratopsidae) skulls from the Upper Cretaceous (Campanian) of Alberta, and an update on the distribution of accessory frill fenestrae in Chasmosaurinae. PeerJ 6, e5194. <https://doi.org/10.7717/peerj.5194>

Campbell, J.A., Ryan, M.J., Schroder-Adams, C.J., Holmes, R.B., Evans, D.C., 2019. Temporal range extension and evolution of the chasmosaurine ceratopsid ‘*Vagaceratops*’ *irvinensis* (Dinosauria: Ornithischia) in the Upper Cretaceous (Campanian) Dinosaur Park Formation of Alberta. Vertebrate Anatomy Morphology Palaeontology 7, 83–100. <https://doi.org/10.18435/vamp29356>

Campione, N. E., K. S. Brink, E. A. Freedman, C. T. McGarrity, and D. C. Evans. 2013. ‘*Glishades ericksoni*’, an indeterminate juvenile hadrosaurid from the Two Medicine Formation of Montana: implications for hadrosauroid diversity in the latest Cretaceous (Campanian–Maastrichtian) of western North America. Palaeobiodiversity and Palaeoenvironments 93:65–75.

Currie, P. J., and D. A. Russell. 2005. The geographic and stratigraphic distribution of articulated and associated dinosaur remains; pp. 537–569 in P. J. Currie and E. B. Koppelhus (eds.), Dinosaur Provincial Park: A Spectacular Ancient Ecosystem Revealed. Indiana University Press, Bloomington, Indiana.

Diem, S., and J. D. Archibald. 2005. Range extension of southern chasmosaurine ceratopsian dinosaurs into northwestern Colorado. Journal of Paleontology 79:251–258.

Dodson, P., C. A. Forster, and S. D. Sampson. 2004. Ceratopsidae; pp. 494–513 in D. B. Weishampel, P. Dodson, and H. Osmolska (eds.), The Dinosauria. University of California Press, Berkeley.

Eberth, D. A. 2005. The Geology; pp. 54–82 in P. J. Currie and E. B. Koppelhus (eds.), Dinosaur Provincial Park: A Spectacular Ancient Ecosystem Revealed. Indiana University Press, Bloomington, Indiana.

Eberth, D. A. 2011. A revised chronostratigraphy for Dinosaur Provincial Park, Alberta, Canada;in Speaker Series, Royal Tyrrell Museum, Drumheller, Alberta.

Farke, A. A. 2006. Morphology and ontogeny of the cornual sinuses in chasmosaurine dinosaurs (Ornithischia: Ceratopsidae). Journal of Paleontology 80:780–785.

Fassett, J. E. 2009. New geochronologic and stratigraphic evidence confirms the Paleocene age of the dinosaur-bearing Ojo Alamo Sandstone and Animas Formation in the San Juan Basin, New Mexico and Colorado. Palaeontologia Electronica 12:1–150.

Fassett, J. E., and J. S. Hinds. 1971. Geology and fuel resources of the Fruitland Formation and Kirtland Shale of the San Juan Basin, New Mexico and Colorado. United States Geological Survey Professional Paper 676:1–76.

Fassett, J. E., and M. B. Steiner. 1997. Precise age of C33N-C32R magnetic-polarity reversal, San Juan Basin, New Mexico and Colorado. New Mexico Geological Society Guidebook 48:239–247.

Fastovsky, D. E., D. B. Weishampel, M. Watabe, R. Barsbold, K. H. Tsogtbaatar, and P. Narmandakh. 2011. A nest of *Protoceratops andrewsi* (Dinosauria, Ornithischia). Journal of Paleontology 85:1035–1041.

Forster, C. A., P. C. Sereno, T. W. Evans, and T. Rowe. 1993. A complete skull of *Chasmosaurus mariscalensis* (Dinosauria: Ceratopsidae) from the Aguja Formation (late Campanian) of West Texas. Journal of Vertebrate Paleontology 13:161–170.

Fowler, D. W., H. N. Woodward, E. A. Freedman, P. L. Larson, and J. R. Horner. 2011. Reanalysis of *Raptorex kriegsteini*: A Juvenile Tyrannosaurid Dinosaur from Mongolia. PLoS ONE 6:e21376.

Fowler, D.W., 2017. Revised geochronology, correlation, and dinosaur stratigraphic ranges of the Santonian–Maastrichtian (Late Cretaceous) formations of the Western Interior of North America. PLOS ONE 12, e0188426. <https://doi.org/10.1371/journal.pone.0188426>

Fowler, D.W., Freedman Fowler, E.A., 2017. The oldest “*Chasmosaurus*”? A new skull from the Judith River Formation of Montana, in: Society of Vertebrate Paleontology, Annual Meeting Abstracts. p. 114.

Gilmore, C. W. 1919. Reptilian faunas of the Torrejon, Puerco, and underlying Upper Cretaceous formations of San Juans County, New Mexico. United States Geological Survey Professional Paper 119:1–71.

Gilmore, C. W. 1946. Reptilian fauna of the North Horn Formation of central Utah. United States Geological Survey Professional Paper 210-C:29–53.

Godfrey, S. J., and R. B. Holmes. 1995. Cranial morphology and systematics of *Chasmosaurus* (Dinosauria: Ceratopsidae) from the Upper Cretaceous of western Canada. Journal of Vertebrate Paleontology 15:726–742.

Goodwin, M. B., W. A. Clemens, J. R. Horner, and K. Padian. 2006. The smallest known *Triceratops* skull: new observations on ceratopsid cranial anatomy and ontogeny. Journal of Vertebrate Paleontology 26:103–112.

Hatcher, J. B., O. C. Marsh, and R. S. Lull. 1907. The Ceratopsia. United States Geological Monograph 49:1–300.

Holmes, R. B., C. A. Forster, M. J. Ryan, and K. M. Shepherd. 2001. A new species of *Chasmosaurus* (Dinosauria: Ceratopsia) from the Dinosaur Park Formation of southern Alberta. Canadian Journal of Earth Sciences 38:1423–1438.

Horner, J. R., and M. B. Goodwin. 2006. Major cranial changes during *Triceratops* ontogeny. Proceedings of the Royal Society B 273:2757–2761.

Horner, J. R., and M. B. Goodwin. 2008. Ontogeny of cranial epi-ossifications in *Triceratops*. Journal of Vertebrate Paleontology 28:134–144.

Horner, J. R., D. J. Varricchio, and M. B. Goodwin. 1992. Marine transgressions and the evolution of Cretaceous dinosaurs. Nature 358:59–61.

Hunt, A. P., and S. G. Lucas. 1992. Stratigraphy, paleontology, and age of the Fruitland and Kirtland Formations (Upper Cretaceous), San Juan Basin, New Mexico. New Mexico Geological Society Guidebook 43:217–239.

Hunt, A. P., and S. G. Lucas. 2003. Origin and stratigraphy of historic dinosaur quarries in the Upper Cretaceous Fruitland Formation of the Fossil Forest Research Natural Area, northwestern New Mexico. New Mexico Geological Society Guidebook, 54th Field Conference, Geology of the Zuni Plateau:383–388.

Kuiper, K. F., A. L. Deino, F. J. Hilgen, W. Krijgsman, P. R. Renne, and J. R. Wijbrans. 2008. Synchronizing rock clocks of Earth history. Science 320:500–504.

Lambe, L. M. 1902. New genera and species from the Belly River Series (mid-Cretaceous). Contributions to Canadian Paleontology 3:25–81.

Lambe, L. M. 1914a. On the fore-limb of a carnivorous dinosaur from the Belly River Formation of Alberta, and a new genus of Ceratopsia from the same horizon, with remarks on the integument of some Cretaceous herbivorous dinosaurs. The Ottawa Naturalist 27:130–135.

Lambe, L. M. 1914b. On *Gryposaurus notabilis*, a new genus and species of trachodont dinosaur from the Belly River Formation of Alberta, with a description of the skull of *Chasmosaurus belli*. The Ottawa Naturalist 27:145–155.

Lambe, L. M. 1915. On *Eoceratops canadensis*, gen. nov., with remarks on the other genera of Cretaceous horned dinosaurs. Geological Survery of Canada Museum Bulletin 12:1–49.

Langston, W., Jr. 1959. *Anchiceratops* from the Oldman Formation of Alberta. Natural History Papers, National Museum of Canada 3:1–11.

Lawson, D. A. 1976. *Tyrannosaurus* and *Torosaurus*, Maestrichtian dinosaurs from Trans-Pecos, Texas. Journal of Paleontology 50:158–164.

Lehman, T. M. 1989. *Chasmosaurus mariscalensis*, n. sp., a new ceratopsian dinosaur from Texas. Journal of Vertebrate Paleontology 9:137–162.

Lehman, T. M. 1990. The ceratopsian subfamily Chasmosaurinae: sexual dimorphism and systematics; pp. 211–229 in K. Carpenter and P. J. Currie (eds.), Dinosaur Systematics Approaches and Perspectives. Cambridge University Press, Cambridge.

Lehman, T. M. 1993. New data on the ceratopsian dinosaur *Pentaceratops sternbergii* Osborn from New Mexico. Journal of Paleontology 67:279–288.

Lehman, T. M. 1998. A gigantic skull and skeleton of the horned dinosaur *Pentaceratops sternbergi* from New Mexico. Journal of Paleontology 72:894–906.

Lehman, T. M. 2007. Growth and population age structure in the horned dinosaur *Chasmosaurus*; pp. 259–317 in K. Carpenter (ed.), Horns and Beaks: Ceratopsian and Ornithopod Dinosaurs. Indiana University Press, Bloomington.

Lehman, T. M., and A. B. Busbey III. 2007. Big Bend Field Trip Guidebook. Society of Vertebrate Paleontology, 67th Annual Meeting Field Trip Guidebook:1–69.

Lehman, T. M., F. McDowell, and J. Connelly. 2006. First isotopic (U-Pb) age for the Late Cretaceous *Alamosaurus* vertebrate fauna of West Texas, and its significance as a link between two faunal provinces. Journal of Vertebrate Paleontology 26:922–928.

Lehman, T.M., Wick, S.L., Wagner, J.R., 2016. Hadrosaurian dinosaurs from the Maastrichtian Javelina Formation, Big Bend National Park, Texas. Journal of Paleontology 90, 333–356. <https://doi.org/10.1017/jpa.2016.48>

Lehman, T.M., Wick, S.L., Barnes, K.R., 2017. New specimens of horned dinosaurs from the Aguja Formation of West Texas, and a revision of *Agujaceratops*. Journal of Systematic Palaeontology 15, 641–674. <https://doi.org/10.1080/14772019.2016.1210683>

Loewen, M. A., A. A. Farke, S. D. Sampson, M. A. Getty, E. K. Lund, and P. M. O'Connor. 2013. Ceratopsid dinosaurs from the Grand Staircase of southern Utah; pp. 488–503 in A. L. Titus and M. A. Loewen (eds.), At the Top of the Grand Staircase: the Late Cretaceous of Southern Utah. Indiana University Press, Bloomington.

Loewen, M. A., S. D. Sampson, E. K. Lund, A. A. Farke, M. C. Aguillón-Martínez, C. A. de Leon, R. A. Rodríguez-de la Rosa, M. A. Getty, and D. A. Eberth. 2010. Horned dinosaurs (Ornithischia: Ceratopsidae) from the Upper Cretaceous (Campanian) Cerro del Pueblo Formation, Coahila, Mexico; pp. 99–116 in M. J. Ryan, B. J. Chinnery-Allgeier, and D. A. Eberth (eds.), New Perspectives on Horned Dinosaurs: The Royal Tyrrell Museum Ceratopsian Symposium. Indiana University Press, Bloomington.

Longrich, N. R. 2010. *Mojoceratops perifania*, a new chasmosaurine ceratopsid from the late Campanian of western Canada. Journal of Paleontology 84:681–694.

Longrich, N. R. 2011. *Titanoceratops ouranos*, a giant horned dinosaur from the late Campanian of New Mexico. Cretaceous Research 32:264–276.

Longrich, N. R. 2014. The horned dinosaurs *Pentaceratops* and *Kosmoceratops* from the upper Campanian of Alberta and implications for dinosaur biogeography. Cretaceous Research 51:292–308.

Longrich, N., 2015. Systematics of *Chasmosaurus* - new information from the Peabody Museum skull, and the use of phylogenetic analysis for dinosaur alpha taxonomy. F1000Research 4. <https://doi.org/10.12688/f1000research.7573.1>

Lucas, S. G., A. P. Hunt, and R. M. Sullivan. 2006. Stratigraphy and age of the Upper Cretaceous Fruitland Formation, west-central San Juan Basin, New Mexico. New Mexico Museum of Natural History and Science Bulletin 35:1–6.

Lucas, S. G., R. M. Sullivan, and A. P. Hunt. 2006. Re-evaluation of *Pentaceratops* and *Chasmosaurus* (Ornithischia: Ceratopsidae) in the Upper Cretaceous of the Western Interior. New Mexico Museum of Natural History and Science Bulletin 35:367–370.

Lucas, S. G., R. M. Sullivan, S. M. Cather, S. E. Jasinski, D. W. Fowler, A. B. Heckert, J. A. Spielmann, and A. P. Hunt. 2009. No definitive evidence of Paleocene dinosaurs in the San Juan Basin. Palaeontologia Electronica 12:1–10.

Lull, R. S. 1933. A revision of the Ceratopsia or horned dinosaurs. Peabody Museum of Natural History Bulletin 3:1–175.

Maidment, S. C. R., and P. M. Barrett. 2011. A new specimen of *Chasmosaurus belli* (Ornithischia: Ceratopsidae), a revision of the genus, and the utility of postcrania in the taxonomy and systematics of ceratopsid dinosaurs. Zootaxa 2963:1–47.

Mallon, J. C., D. C. Evans, M. J. Ryan, and J. S. Anderson. 2012. Megaherbivorous dinosaur turnover in the Dinosaur Park Formation (upper Campanian) of Alberta, Canada. Palaeogeography, Palaeoclimatology, Palaeoecology 350–352:124–138.

Mallon, J. C., R. B. Holmes, D. A. Eberth, M. J. Ryan, and J. S. Anderson. 2011. Variation in the skull of *Anchiceratops* (Dinosauria: Ceratopsidae) from the Horseshoe Canyon Formation (Upper Cretaceous) of Alberta. Journal of Vertebrate Paleontology 31:1047–1071.

Mallon, J. C., R. B. Holmes, J. S. Anderson, A. A. Farke, D. C. Evans, and H.-D. Sues. 2014. New information on the rare horned dinosaur *Arrhinoceratops brachyops* (Ornithischia: Ceratopsidae) from the Upper Cretaceous of Alberta, Canada. Canadian Journal of Earth Sciences 51:618–634.

Min, K., R. Mundil, P. R. Renne, and K. R. Ludwig. 2000. A test for systematic errors in 40Ar/39Ar geochronology through comparison with U/Pb analysis of a 1.1-Ga rhyolite. Geochimica et Cosmochimica Acta 64:73–98.

Newman, K. R. 1987. Biostratigraphic correlation of Cretaceous–Tertiary boundary rocks, Colorado to San Juan Basin, New Mexico; pp. 151–163 in J. E. Fassett and J. K. Rigby Jr. (eds.), Cretaceous–Tertiary Boundary in the San Juan and Raton Basins, New Mexico and Colorado. Geological Society of America Special Paper.

O’Sullivan, R. B., and H. M. Beikman. 1963. Geology, Structure, and Uranium Deposits of the Shiprock Quadrangle, New Mexico and Arizona. pp.

O’Sullivan, R. B., G. R. Scott, and J. S. Heller. 1979. Preliminary Geologic Map of the Bisti Trading Post Quadrangle, San Juan County, New Mexico. pp.

Ogg, J. G., L. A. Hinnov, and C. Huang. 2012. Cretaceous; pp. 793–853 in F. M. Gradstein, J. G. Ogg, M. D. Schmitz, and G. M. Ogg (eds.), The Geologic Time Scale 2012. Elsevier, Amsterdam.

Osborn, H. F. 1923. A new genus and species of Ceratopsia from New Mexico, *Pentaceratops sternbergii*. American Museum Novitates 93:1–3.

Rasband, W. S. 1997–2014. ImageJ. U. S. National Institutes of Health, Bethesda, Maryland, USA, http://imagej.nih.gov/ij/.

Roberts, E. M., S. D. Sampson, A. L. Deino, S. A. Bowring, and R. Buchwaldt. 2013. The Kaiparowits Formation: a remarkable record of Late Cretaceous terrestrial environments, ecosystems, and evolution in western North America; pp. 85–106 in A. L. Titus and M. A. Loewen (eds.), At the Top of the Grand Staircase: the Late Cretaceous of Southern Utah. Indiana University Press, Bloomington, Indiana.

Rowe, T., E. H. Colbert, and J. D. Nations. 1980/1981. The occurrence of *Pentaceratops* (Ornithischia: Ceratopsia) with a description of its frill; pp. 29–48 in S. G. Lucas, J. K. Rigby Jr., and B. S. Kues (eds.), Advances in San Juan Basin Paleontology. University of New Mexico Press, Albuquerque.

Rowe, T., R. L. Cifelli, T. M. Lehman, and A. Weil. 1992. The Campanian Terlingua Local Fauna, with a summary of other vertebrates from the Aguja Formation, Trans-Pecos Texas. Journal of Vertebrate Paleontology 12:472–493.

Rozhdestvensky, A. K. 1965. Growth changes in Asian dinosaurs and some problems of their taxonomy. Paleontologischeskii Zhurnal 3:95–109.

Ryan, M. J., and D. C. Evans. 2005. Ornithischian Dinosaurs. Dinosaur Provincial Park: A Spectacular Ancient Ecosystem Revealed:312–348.

Sampson, S. D., M. A. Loewen, A. A. Farke, E. M. Roberts, C. A. Forster, J. A. Smith, and A. L. Titus. 2010. New horned dinosaurs from UTah provide evidence for intracontinental dinosaur endemism. PLoS ONE 5:e12292.

Scannella, J. B., and J. R. Horner. 2010. *Torosaurus* Marsh, 1891, is *Triceratops* Marsh, 1889 (Ceratopsidae: Chasmosaurinae): synonymy through ontogeny. Journal of Vertebrate Paleontology 30:1157–1168.

Scannella, J. B., D. W. Fowler, M. B. Goodwin, and J. R. Horner. 2014. Evolutionary trends in *Triceratops* from the Hell Creek Formation, Montana. Proceedings of the National Academy of Sciences 111:10245–10250.

Scott, G. R., R. B. O’Sullivan, and J. S. Heller. 1979. Preliminary Geologic Map of the Burnham Trading Post Quadrangle, San Juan County, New Mexico. pp.

Sternberg, C.M. 1919. Field notes, Summer 1919. Unpublished field notes archieved at the Canadian Museum of Nature, Ottawa, Canada.

Sternberg, C. M. 1940. Ceratopsidae from Alberta. Journal of Paleontology 14:468–480.

Sternberg, C. M. 1950. Steveville - west of the fourth meridian, Alberta. Geological Survey of Canada Topographic Map A.

Sullivan, R. M. 2006. Ah-shi-sle-pah Wilderness Study Area (San Juan Basin, New Mexico): a paleontological (and historical) treasure and resource. New Mexico Museum of Natural History and Science Bulletin 34:169–174.

Sullivan, R. M., and S. G. Lucas. 2006. The pachycephalosaurid dinosaur *Stegoceras validum* from the Upper Cretaceous Fruitland Formation, San Juan Basin, New Mexico. New Mexico Museum of Natural History and Science Bulletin 35:329–330.

Sullivan, R. M., and S. G. Lucas. 2011. Charles Hazelius Sternberg and his San Juan Basin Cretaceous dinosaur collections: correspondence and photographs (1920–1925). New Mexico Museum of Natural History and Science Bulletin 53:429–471.

Sullivan, R. M., S. G. Lucas, and D. R. Braman. 2005. Dinosaurs, pollen, and the Cretaceous-Tertiary boundary in the San Juan Basin, New Mexico. New Mexico Geological Society, 56th Field Conference Guidebook, Geology of the Chama Basin:395–407.

Tanke D. H. 2005. Identifying lost quarries. In: Currie PJ, Koppelhus EB eds. *Dinosaur Provincial Park: a spectacular ancient ecosystem revealed*. Bloomington, Indiana: Indiana University Press, 34–53.

Tanke, D. H. 2010. Ceratopsian discoveries and work in Alberta, Canada: historical review and census; pp. 488 (CD-ROM) in M. J. Ryan, B. J. Chinnery-Allgeier, and D. A. Eberth (eds.), New Perspectives on Horned Dinosaurs: The Royal Tyrrell Museum Ceratopsian Symposium. Indiana University Press, Bloomington.

Tanke, D. H., and B. M. Rothschild. 2002. Dinosores: an annotated bibliography of dinosaur paleopathology and related topics - 1838–2001. New Mexico Museum of Natural History and Science Bulletin 20:1–97.

Weide, D. L., G. B. Schneider, J. W. Mytton, and G. R. Scott. 1979. Geologic map of the Pueblo Bonito Quadrangle, San Juan County, New Mexico, Report 1119.

Wick, S. L., and T. M. Lehman. 2013. A new ceratopsian dinosaur from the Javelina Formation (Maastrichtian) of West Texas and implications for chasmosaurine phylogeny. Naturwissenschaften 100:667–682.

Wiman, C. 1930. Uber Ceratopsia aus der oberen Kreide in New Mexico. Nova Acta Regiae Societatis Scientarum Upsaliensis 4-7:1–19.

Wolberg, D. L., J. P. Hall, and D. Bellis. 1988. First record of dinosaur footprints from the Fruitland Formation, San Juan County, New Mexico. New Mexico Bureau of Mines and Mineral Resources, Bulletin 122:33–34.Breyer, J. A., A. B. Busbey III, R. E. Hanson, K. E. Befus, W. R. Griffin, U. S. Hargrove, and S. C. Bergman. 2007. Evidence for Late Cretaceous Volcanism in Trans-Pecos Texas. The Journal of Geology 115:243–251.
